# Phytolith assemblages from palm leaves and palm-leaf manuscripts: what is the difference and what it could mean?

**DOI:** 10.3389/fpls.2024.1482790

**Published:** 2025-01-14

**Authors:** Anastasia Poliakova, Giovanni Ciotti, Agnieszka Helman-Wazny, Jörg Fromm

**Affiliations:** ^1^ Centre for the Study of Manuscript Cultures, University of Hamburg, Hamburg, Germany; ^2^ Cluster of Excellence “Understanding Written Artefacts”, University of Hamburg, Hamburg, Germany; ^3^ Institute for Chemistry, University of Hamburg, Hamburg, Germany; ^4^ Department of History and Cultures, University of Bologna, Bologna, Italy; ^5^ Federal Institute for Materials Research and Testing (BAM), Berlin, Germany; ^6^ Faculty of Journalism, Information and Book Studies, University of Warsaw, Warsaw, Poland; ^7^ Institute for Wood Science, University of Hamburg, Hamburg, Germany

**Keywords:** phytoliths, Arecaceae, palaeoecology, material codicology, palm-leaf manuscripts

## Abstract

We studied freshly collected, dried and herbarized leaf fragments of two palms, namely *Borassus flabellifer* L. and *Corypha umbraculifera* L., most commonly used for palm-leaf manuscript (PLM) production in South (S) and Southeast Asia (SE) in order to reveal differences in their phytolith assemblages. For each of the two palms, 25 leaf samples were taken from the two Indian states of Kerala and Tamil Nadu. Dried leaf material was obtained from the fresh one by drying the leaves in air. Herbarium samples were obtained from two independent herbaria, specimen origin comprises S and SE Asia with the main focus on South India and Sri Lanka. Additionally, 25 manuscripts made of *Borassus flabellifer* leaves and 25 manuscripts made of *Corypha umbraculifera* leaves were investigated for phytoliths. All manuscripts are preliminary dated back to between the 16^th^ and the beginning of the 20^th^ century CE; most of them assumedly were produced in S India (Tamil Nadu and Kerala), Sri Lanka, Burma or Indonesia. Phytolith assemblages significantly differed between fresh, dry and herbarized palm leaves in comparison to PLM material, both qualitatively and quantitatively (mean r^2^ = - 0.61 ± 9.3 for *Borassus* samples and r^2^ = - 0.75 ± 5.3 for *Corypha* samples, at p < 0.001). Fifty-three phytolith types described for PLM material were not observed in any of the fresh, dry or herbarized palm-leaf samples. Geographical analysis of PLM-specific phytoliths suggests that the combination of those phytoliths could be region-related. In this paper, we prove that the methods of palaeoecological reconstructions based on detailed microscopy of the PLMs surface and phytolith analysis applied in combination with methods of mathematical and computer data analysis can contribute to answer the questions posed by material codicology by revealing lost manuscript production recipes and by studying manuscript provenance in terms of the geographical origin of the artefacts. Our approach can potentially open a new perspective for palaeoecological studies expanding their traditional scope and making them applicable to a new research field.

## 1 Introduction

With recent progress in phytolith studies, opal silica bodies from plants have been widely utilized in various archaeological (e.g., Albert and Weiner, 2001; Piperno, 2006, Albert and Weiner, 2001; Madella et al., 2009, Madella et al., 2013), palaeoclimatic (e.g., Mulholland, 1989; Wang and Lu, 1993; Gao et al., 2018; Wang et al., 2019, Wang et al., 2022), and worldwide palaeoenvironmental and palaeovegetation studies (e.g., Gao et al., 2021; Liu et al., 2023). However, due to phytolith translocation and corrosion, phytolith assemblages sometimes fail to accurately reflect the plant community and climatic conditions (e.g., Fishkis et al., 2010; Dan et al., 2011; Zuo et al., 2014).

Phytoliths extracted from different sources such as soil, sediments, and other geological sequences have proven to be significant proxies for a variety of Late Quaternary reconstructions (see Introduction in Lu et al., 2006). Their preservation, distribution, and abundance in sediments are sensitive to environmental conditions (e.g., Mulholland, 1989; Madella, 1997; Liu et al., 2023). Phytolith assemblages, however, do not necessarily indicate that plants, whose phytoliths are found together in the same layer, coexisted. Numerous archaeological investigations have demonstrated the applicability of phytolith analysis to identify plant usage for various purposes, such as fuel (Albert and Weiner, 2001) and food, as evidenced by studies of grinding stones (Radomski and Neumann, 2011) and other food-related artifacts including pottery (Piperno, 2006, Piperno, 2009). Dietary studies involving phytolith analysis have examined dinosaur coprolites (Piperno and Sues, 2005; Prasad, 2005), modern primate feces (Phillips and Lancelotti, 2014), and dental calculus of early hominins (e.g., Fox et al., 1996; Henry and Piperno, 2008; Power et al., 2018).

In this paper, we propose to extend the classical use of phytolith analysis to address research questions in material codicology and in the study of old manuscripts. We demonstrate that phytolith assemblages described from the surface of palm-leaf manuscripts (PLMs) can aid in reconstructing the plants historically used in PLM production in South (S) and Southeast (SE) Asia (Agrawal, 1984; Sah, 2002; Henderson, 2009; Wiland et al., 2022). Despite extensive literature on the conservation and restoration of PLMs (we analyzed so far approximately 300 monographs, research articles, and short communications; Poliakova et al., in preparation), the details of PLM production, regional peculiarities and especially historical changes, remain largely poorly described. To the best of our knowledge, no studies have addressed these aspects, and historical practices of PLM production, often are essentially lost in the regions under investigation. Our research presented here aims to fill this large knowledge gap by providing evidence that palaeoecological methods, such as phytolith analysis combined with high-resolution microscopy widely used for reconstructing past vegetation patterns, environmental conditions, and land use practices - can be adapted to the study of material codicology and old PLM analysis. We selected leaf material and PLMs from *Borassus flabellifer* L. and *Corypha umbraculifera* L. - two palm species most commonly used as writing supports in S and SE Asia (Harinarayana, 1995; Freeman, 2005; Wilson and Rice, 2019; Nishanthi and Wijayasundara, 2022).

This study aims to (1) Coherently study and compare opal phytolith assemblages from the unprocessed material, i.e., fresh, dry, and herbarized palm leaf samples from the two species. (2) Identify any differences between the phytolith assemblages of these materials and those obtained from PLMs. (3) Compare phytolith assemblages from the inner leaf tissues of all research material with those from the surfaces of the same material, focusing on exotic phytoliths found on the surfaces of PLMs in order to demonstrate that methods of palaeoecological reconstructions can help identify plants, those in addition to the palms are used in the PLM production process. (4) Study possible differences in the phytolith assemblages described from the surface of PLMs of different geographical origin. (5) Study the role of random phytolith contamination of all types of research material and to evaluate, to which extent the environmental contamination influences the accuracy of the phytolith analysis of palm samples from S and SE Asia.

## 2 Materials and methods

### 2.1 Fresh and dry palm leaf samples of *Borassus flabellifer* and *Corypha umbraculifera*

Leaf fragments of two palm species, *Borassus flabellifer* L. and *Corypha umbraculifera* L., commonly used for manuscript production (Suvatabandhu, 1962; Agrawal, 1984; Sah, 2002; Padmakumar and Sreekumar, 2003; Kumar et al., 2009), were used for this study. Samples from freshly cut palm leaves, dried and dead leaves, herbarium specimens, and PLMs of both species were included. Freshly cut leaves of *Borassus* and *Corypha* were collected in Tamil Nadu (22 samples) by members of the Ecology Department of the French Institute of Puducherry (India) in May 2024 and by the first author in June and July 2024 in Tamil Nadu (7 samples) and Kerala (19 samples), South India. A full list of the fresh and dry palm leaf samples, including coordinates of collection sites, is provided in **Supplementary Material S1**. The collected material was divided into two portions: one portion was in order to avoid mould frozen at -5°C to be studied as *Borassus* fresh leaf samples (BF) and *Corypha* fresh leaf samples (CF), and the other portion was air-dried and studied as *Borassus* dry leaf samples (BD) and *Corypha* dry leaf samples (CD), respectively. Fresh material represents a modern and unprocessed palm leaves that have on the surface only natural present-day contamination usual in the given environment. Dry leave samples (also modern and unprocessed) in comparison to the fresh ones bare on their surface more cotemporally contamination and dust collected as a result of the process of drying in the open air. No freezing was applied to the dried material.

### 2.2 Herbarium samples of *Borassus flabellifer* and *Corypha umbraculifera*

**Figure 1:**
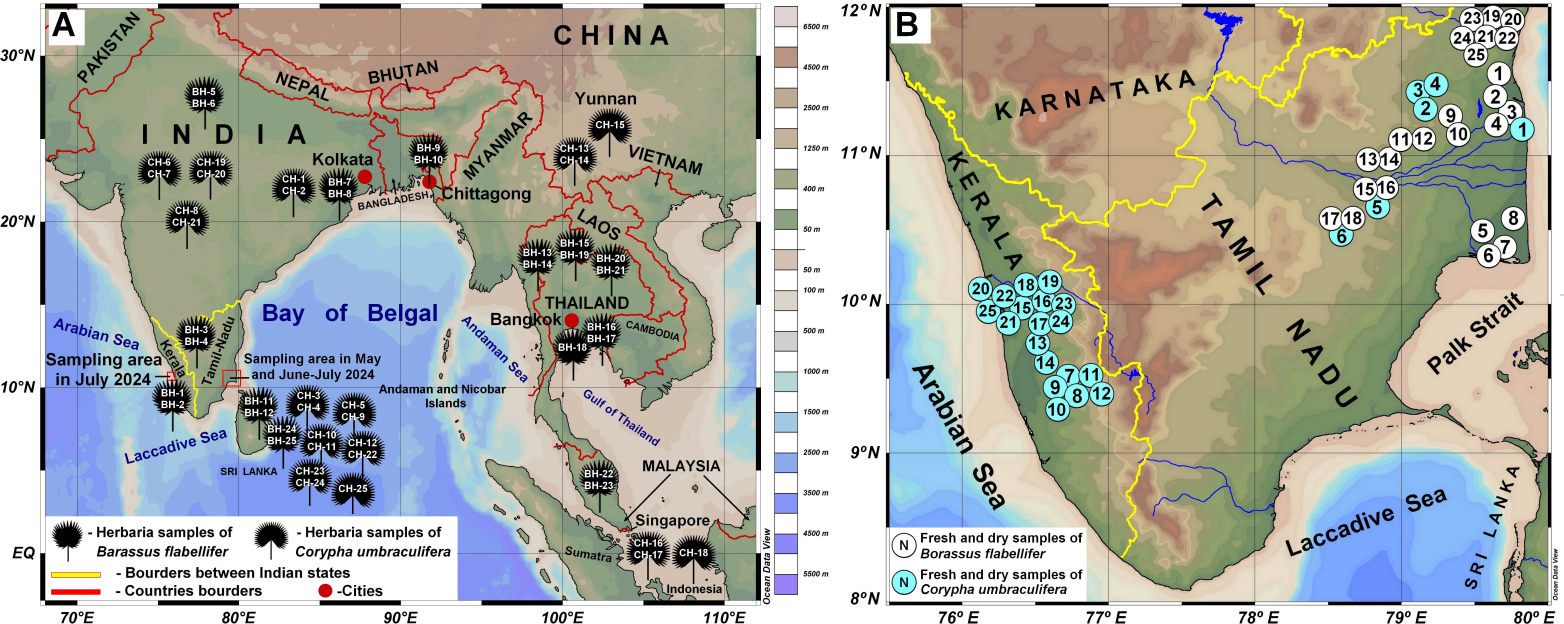
Origin or the research material used in this study. **(A)** Herbarium material collected from the University of Göttingen (Germany, January 2022) and Royal Botanic Gardens, Kew (UK; June 2023). Material origin is mapped according to the information indicated at the collection labels and as given in the personal communication with curators. **(B)** Fresh and dry samples collected in India, in the states of Tamil Nadu and Kerala in May-July 2024, see **Supplementary Material S1** for details on exact sampling locations given as numbers. Maps were created using QGIS Development Team (2009) and the Ocean Data View software and a base ground map (Schlitzer, 2015), available from: http://odv.awi.de.

Herbarium samples (1.5-5 mm x 2-5 mm) were obtained from collections of the University of Göttingen (GOET, Germany; collected in January 2022) and Royal Botanic Gardens, Kew (Kew Gardens, UK; collected in June 2023). Details of the herbarium sample origins are provided in **Figure 1** and **Supplementary Material S2**. When sampling from the same herbarium specimen, material was taken from different leaves. We aimed for possibly older material (collected in the 1950s and earlier) to compare with palm manuscript samples. Additionally, some samples collected from the herbarium aged 1970s - 2000s were included for comparison (See **Supplementary Material S2**).

### 2.3 Palm-leaf manuscripts samples

Palm-leaf manuscripts were sampled from the collections of the Centre for the Study of Manuscript Cultures of the University of Hamburg (CMSC; 3 *Corypha* manuscripts), the State and University Library of Hamburg (SUB UHH; 17 *Borassus flabellifer* and 12 *Corypha umbraculifera* manuscripts, palm species identified microscopically), and the Archive of the École Française D’extrême-Orient in Puducherry, India (EFEO; 3 *Borassus* and 4 *Corypha* manuscripts; see **Table 1**). Additionally, 5 *Borassus* and 6 *Corypha* manuscripts from private collectors were sampled with the owners’ permission. When allowed, manuscripts were sampled by cutting 1-2 mm strips from the margins, trimming from destroyed edges, or from binding holes if the manuscript was intact and preserved well, or if it was covered with lacquer or natural lac. To minimize damage, fragments that had fallen apart and bore no text were collected when possible; in each case, only the minimum material needed for studies was collected. To study possible geographical variations in phytolith assemblages in the manuscript samples, site assignments were based on the philological analysis of the manuscripts. A first criterion is the identifying the script, which by itself pins the origin of the manuscripts to specific macro-regions, such as Tamil Nadu, Kerala, Sri Lanka, Burma/Myanmar. Furthermore, if available, scribal colophons, i.e., statements directly composed by the scribes of each individual manuscript, were investigated, with a particular attention to the mention of place names, either those explicitly given as the place where the manuscripts were copied or those that are part of the name of the scribe. Although place names of either category do not by themselves assure the identification of the place of origin of the leaves used for producing a given manuscript, they offer nevertheless a reliable starting point. In fact, one may consider that *Borassus* grows widely in the areas in question and manuscripts were most probably produced with locally available leaves. The situation may be different for manuscript made of *Corypha* leaves. One can confidently observe how *Corypha* leaves were traded to north India and Central Asia for several centuries since at least the first millennium CE, but also from Sri Lanka to Tamil Nadu. Furthermore, manuscripts are dated either based on the explicit dates given by the scribes, or on palaeographical assumptions. In any case, given the majority of the dates found in colophons (for Tamil Nadu, see Ciotti and Franceschini, 2016; Franceschini, 2022), it is reasonable to assume that most of the extant palm-leaf manuscripts in the areas taken into consideration for this article date back to the 19th century, with a few whose date can stretch back to the 16th century. In the absence of colophons, the preliminary geographical origin was assigned based on philological analysis of the script and writing style. Text and script analysis were performed at CSMC UHH and at the University of Bologna.

**Table 1:** Palm-leaf manuscript microsamples collected for phytolith studies in 2022-2024.

*Borassus flabellifer*				
Sample number	Sample code	Material origin	Age (yr or century)/ manuscript origin	Sampling date
BM-1	Cod. Palmbl. I 5 (35.3005)	SUB Hamburg	16 cent. / Tamil Nadu, India	23.03.2023
BM-2	Cod. Palmbl. II 209 (35.3209)	SUB Hamburg	1885 / Tamil Nadu, India	08.04.2023
BM-3	Cod. Palmbl. I 33 (35.3033)	SUB Hamburg	1802 / Tamil Nadu, India	19.04.2023
BM-4	Cod. Palmbl. I 24 (35.3024)	SUB Hamburg	19 cent. / Tamil Nadu, India	26.04.2023
BM-5	Cod. Palmbl. I 170 (35.3170)	SUB Hamburg	19 cent. / Tamil Nadu, India	26.04.2023
BM-6	Cod. Palmbl. I 51 (35.3051)	SUB Hamburg	1840 / Tamil Nadu, India	26.04.2023
BM-7	Cod. Palmbl. II 205 (35.3205)	SUB Hamburg	19 cent. / Tamil Nadu, India	05.05.2023
BM-8	Cod. Palmbl. II 224 (35.24)	SUB Hamburg	19 cent. / Tamil Nadu, India	05.05.2023
BM-9	Cod. Palmbl. I 28 (35.3028)	SUB Hamburg	19 cent. / Tamil Nadu, India	05.05.2023
BM-10	Cod. Palmbl. I 11 (35.3011)	SUB Hamburg	19 cent. / Tamil Nadu, India	10.05.2023
BM-11	Cod. Palmbl. I 169 (35.3169)	SUB Hamburg	19 cent. / Tamil Nadu, India	10.05.2023
BM-12	Cod. Palmbl. I 112 (35.3112)	SUB Hamburg	19 cent. / Tamil Nadu, India	10.05.2023
BM-13	Cod. Palmbl. III 53 (35.3299)	SUB Hamburg	19 cent. / Tamil Nadu, India	11.05.2023
BM-14	Cod. Palmbl. I 9 (35.3009)	SUB Hamburg	19 cent. / Tamil Nadu, India	11.05.2023
BM-15	Cod. Palmbl. I 110 (35.3110)	SUB Hamburg	19 cent. / Tamil Nadu, India	11.05.2023
BM-16	CSMC-Malik-2	A. Malik’s manuscript collection	19 cent. / Kerala, India	17.05.2023
BM-17	UHH-HB-Fromm	From Prof. J. Fromm	Modern / Myanmar	07.08.2023
BM-18	CSMC-Rene-Teigeler-29	R. Teigeler’s collection	19-20 cent. / Indonesia, Bali	13.09.2023
BM-19	CSMC-Rene-Teigeler-32	R. Teigeler’s collection	19-20 cent. / Indonesia, Lombok	13.09.2023
BM-20	Cod. Orient 286a	SUB Hamburg	19-20 cent. / Tamil Nadu, India	14.09.2023
BM-21	UHH-PCL-Boye	From Mr. S. Boie	20 cent. / Indonesia, Bali	19-20.09.2023
BM-22	Cod. Palmbl. 35.3366	SUB Hamburg	19-20 cent. / Tamil Nadu, India	20.09.2023
BM-23	EO-0137	EFEO Archive, Puducherry	19 cent. / Tamil Nadu, India	27.06.2024
BM-24	EO-0662	EFEO Archive, Puducherry	19 cent. / Tamil Nadu, India	27.06.2024
BM-25	EO-0943	EFEO Archive, Puducherry	19 cent. / Tamil Nadu, India	27.06.2024
*Corypha umbraculifera*				
CM-1	MS-1-2017	UHH CSMC collection	2017 / Sri Lanka	20.03.2023
CM-2	MS-1-2018	UHH CSMC collection	2018 / Kerala, India	20.03.2023
CM-3	MS-1-2014	UHH CSMC collection	2014 / Bali, Indonesia	20.03.2023
CM-4	Cod. Palmbl. II 208 (35.3208)	SUB Hamburg	1577 / Tamil Nadu, India	22.03.2023
CM-5	Cod. Palmbl. III 118 (35.3363)	SUB Hamburg	19 cent. / Tamil Nadu, India	23.03.2023
CM-6	Cod. Palmbl. I 188 (35.3188)	SUB Hamburg	19 cent. / TamilNadu, India	05.05.2023
CM-7	CSMC-Malik-2-1	A. Malik’s manuscript collection	before 19 cent. / Kerala, India	17.05.2023
CM-8	CSMC-Malik-2-2	A. Malik’s manuscript collection	before 19 cent. / Kerala, India	17.05.2023
CM-9	CSMC-Rene-Teigeler-34-1	R. Teigeler’s collection	19-20 cent. / Burma	02.08.2023
CM-10	CSMC-Rene-Teigeler-34-2	R. Teigeler’s collection	19-20 cent. / Burma	08.08.2023
CM-11	CSMC-Rene-Teigeler-extra	R. Teigeler’s collection	NA / Bali, Indonesia	08.08.2023
CM-12	CSMC-Rene-Teigeler-33	R. Teigeler’s collection	NA / Java; Originally Sri Lanka	11.08.2023
CM-13	Cod. Palmbl. 35.3194	SUB Hamburg	19-20 cent. / Tamil Nadu, India	13.09.2023
CM-14	Cod. Palmbl. 35.3192	SUB Hamburg	19-20 cent. / Tamil Nadu, India	13.09.2023
CM-15	Cod. Palmbl. 35.3249	SUB Hamburg	19-20 cent. / Tamil Nadu, India	15.09.2023
CM-16	Cod. Palmbl. 35.3041	SUB Hamburg	19-20 cent. / Tamil Nadu, India	15-18.09.2023
CM-17	Cod. Palmbl. 35.3044	SUB Hamburg	19-20 cent. / Tamil Nadu, India	20.09.2023
CM-18	Cod. Palmbl. 35.3031	SUB Hamburg	19-20 cent. / Tamil Nadu, India	21.09.2023
CM-19	Cod. Palmbl. 35.3018	SUB Hamburg	19-20 cent. / Tamil Nadu, India	22.09.2023
CM-20	Cod. Palmbl. 35.3032	SUB Hamburg	19-20 cent. / Tamil Nadu, India	25.09.2023
CM-21	Cod. Palmbl. 35.3046	SUB Hamburg	19-20 cent. / Tamil Nadu, India	25.09.2023
CM-22	EO-1612	EFEO Archive, Puducherry	19 cent. / Tamil Nadu, India	24.06.2024
CM-23	EO-1384	EFEO Archive, Puducherry	19 cent. / Tamil Nadu, India	24.06.2024
CM-24	EO-0013	EFEO Archive, Puducherry	19 cent. / Tamil Nadu, India	27.06.2024
CM-25	EO-1454	EFEO Archive, Puducherry	19 cent. / Tamil Nadu, India	02.07.2024

Notes: precise date in yrs CE stays if it was found in the text of the manuscript; ‘before’ stays in case it is was paleographically not possible to detect a creation period of the manuscript more precisely; ‘NA’ indicates that the date of the manuscripts’ creation was not possible to detect.

### 2.4 Phytolith analysis

#### 2.4.1 Samples processing and microscopy

Phytolith extraction followed the method described by Parr et al. (2001) and D’Agostini et al. (2022) with some modifications that considered the specificity of manuscript samples. Every sample was air dried and ashed at 550°C for 3 h in a muffle furnace. After a 12-h cooling period ash was transferred into test tubes to undergo treatment with 10 mL of 10% hydrochloric acid (HCl) for 30 min. One tablet of *Lycopodium clavatum* (number of spores 20,848 ± 1546; Stockmarr, 1971) was added to each sample at the first step of chemical treatment to enable estimation of the phytolith concentration (amount of phytoliths per one ml of studied material) and content (amount of phytoliths per gram of studied material). After centrifugation at 6000 rpm for 5 min, samples were washed twice with distilled water following additional centrifugation after each washing step. Next, samples were treated with 10 mL of 10% hydrogen peroxide (H_2_O_2_) for 2-5 h followed by another 5 min of centrifugation. Thereafter, peroxide was decanted and samples were twice washed with distilled water and centrifuged again. In order to possibly avoid additional erosion and dissolution effect, no vortexing was applied and no strong acids were used. We did not wash nor perform any other sort of cleaning on any of our samples before ashing and wet laboratory processing in order to study the surface environmental contamination in all available samples. Fresh, dry, herbarized and manuscript samples were processed separately, on different days in order to avoid possible cross-contamination.

Residues were kept in the fridge (5°C) in distillate water; slides for light microscopy were prepared with sterile liquid glycerin since that allows to rotate the counted micro-objects and ensures better investigation of the phytolith morphology. Permanent slides of the research material were prepared with glycerin gelatin. Microslides were examined under light microscope at a magnification of x400, x600 and x1000 times. In order to separate phytolith with ambiguous appearance and random mineral particles, polarized light microscopy was applied.

A minimum of 300 phytoliths were counted per sample. All phytoliths greater than 2 µm were photographed, described morphologically and morphometrically, if it was needed for diagnostic purpose. In order to estimate levels of old palm material deterioration (i.e., in herbarized and PLM material), degraded and eroded phytoliths as well as silica sand was counted. All amorphous, rectangular and hexagonal silica fragments of unknown nature as well as phytoliths less than 2 µm in each linear dimension were counted together as silica sand. Sand counting was performed in one observation field at the magnification of x400 and then multiplied by the number of observation fields used for the same phytolith sample. Ashing and all wet laboratory preparations were partly performed at the Department of Palynology and Climate Dynamics, University of Göttingen and partly at the Institute of Plant Sciences and Microbiology (IPM) of the University of Hamburg, Germany.

#### 2.4.2 Phytoliths morphology and identification

All phytolith types described for fresh, dry, herbarized and manuscript material of both investigated palm species, were divided into seven functional groups according to phytolith morphology and morphometry as well as to the most probable source plant group(s) that was determinate, namely (1) Arecaceae, that comprises phytoliths mostly originating from *Borassus* or *Corypha* palm-leaf material, but includes also isolated (i.e., never aggregated) silica bodies of other palms, observed in SEM either strictly on the surface of PLMs or seen as a contamination on the surface of unprocessed leaves. (2) Arecaceae/Zingiberaceae and (3) Arecaceae/Zingiberaceae/Bromeliaceae, seen rarely and randomly on the surface of the unprocessed leaves and often on the PLMs. (4) Musaceae, and (5) Poaceae, mainly registered in the manuscript samples; (6) phytoliths diagnostic for other plants, and (7) non-diagnostic phytoliths. The phytolith morphology and terminology employed here is based on the International Code for Phytolith Nomenclature (ICPN; Neumann et al., 2019), if not stated otherwise.

Phytoliths were identified following morpho-taxonomical guidance of Piperno (2006), Piperno, 2009); Piperno and Pearsall (1998); Pearsall (2011); Chen and Smith (2013), ICPN 2.0 (2019), Piperno and McMichael (2020), and the morphometric studies of Ollendorf (1992); Albert et al. (2009); Fenwick et al. (2011), and Golokhvast et al. (2018). Arecaceae phytoliths were identified following Tomlinson (1961); Sangster and Hodson (1992); Tomlinson et al. (2011); Romain and De Franceschi (2013); Morcote-Ríos et al. (2016); Collura and Neumann (2017); Witteveen et al. (2022); Liu et al. (2023) and literature cited within. In doubtful cases, in order to separate phytoliths from other phytolith types with some similar shape and surface ornamentation, descriptions and illustrations for phytoliths of Orchidaceae (Piperno, 2006; Chen and Smith, 2013; Sharma et al., 2018), Bromeliaceae (Tomlinson, 1969; Piperno, 2006), Cannaceae, Marantaceae, Strelitziaceae, and Zingiberaceae (Kealhofer and Piperno, 1998; Chen and Smith, 2013; Benvenuto et al., 2015; Wang et al., 2022; Dai et al., 2023) and own reference collection material were used.

Musaceae phytoliths were identified as proposed by Mbida Mindzie et al. (2001); Ball et al. (2006); Neumann and Hildebrand (2009) and Chen and Smith (2013). *Cannabis* sp. phytolith assemblage included shapeless, oval, segmented ovals, and club-shaped phytoliths as well as spikes, all ca. 20-50 µm in diameter and ca. 20-70 µm in length as described by Golokhvast et al. (2018); **Table 2**). The grass silica short-cell phytoliths (GSSCP) diagnostic for Poaceae family were classified as described in ICPN 2.0 (2019) and by Dai et al. (2023). When preservation allowed, genera of Poaceae were recognized (e.g., as in Zhao et al., 1998; Yost and Blinnikov, 2011; Gu et al., 2013, Gu et al., 2016; Huan et al., 2015; Tao et al., 2019; Wang et al., 2022; Cordova, 2023). If the phytolith morphology suggest a certain identification but does not look exactly like the reference plant material, phytolith morphotypes are marked with “cf.”

**Table 2:** Table of phytolith morphotypes and inorganic crystals registered in the leaf material of *Borassus flabellifer* and *Corypha umbraculifera*. Codes in brackets indicate contamination.

NN	Code	Phytolith type	Possible source plant(s)
1	Sph_Ech	Spheroid echinate	Arecaceae, Coryphoideae
2	Sph_Ech_Small	Spheroid small echinate (>7 µm)	Arecaceae, Arecoideae
3	Sph_Ech_Large	Spheroid large (>22 µm)	Arecaceae, Coryphoideae, *Borassus* inflorescence
4	Elo_Ech_Att	Elongate echinate (10-12 pro projections, 12-15 µm)	Arecaceae, cf. *Attalea* morphotype
5	Elo_Ech	Elongate echinate	Arecaceae
6	Sto_Iso_Br	Isolated stomata of *Borassus*	Arecaceae, Coryphoideae, *Borassus*
7	Sto_Agr_Br	Stomatal complexes (aggregated stomata of *Borassus*)	Arecaceae, Coryphoideae, *Borassus*
8	Sto_Iso_Cf	Isolated stomata of *Corypha*	Arecaceae, Coryphoideae, *Corypha*
9	Sto_Agr_Cf	Stomatal complexes (aggregated stomata of *Corypha*)	Arecaceae, Coryphoideae, *Corypha*
10	Sph_Psi	Spheroid psilate phytoliths	Arecaceae
11	Sph_Sym	Spheroid verrucate symmetrical	Arecaceae
12	Sph_Asym	Spheroid verrucate asymmetrical	Arecaceae
13	Sph_Acu	Spheroid with acute projections	Arecaceae
14	Sph_Ech	Spheroid echinate with small or “undeveloped” projections	Arecaceae
15	Sph_Fav	Spheroid favose	Arecaceae
16	Asp_Ech	Aspherical echinate	Arecaceae
17	Asp_Ech_rnd	Aspherical echinate with sharp-rounded projections	Arecaceae
18	Asp_Ech_trg	Aspherical echinate with roundish-triangulate projections	Arecaceae
19	Con	Conical phytoliths of Arecaceae	Arecaceae
20	(Con_Ech)	Conical echinate phytoliths (6-8 projections)	Arecaceae, cf. *Bactris* morphotype
21	(Con_Tab)	Conical tabular	Arecaceae, cf. *Bactris simplicifrons* morphotype
22	Ren_Ech	Reniform echinate phytoliths	Arecaceae
23	Ren_ver	Reniform verrucate phytoliths	Arecaceae
24	Ren_Psi	Reniform psilate phytoliths	Arecaceae
25	Ren_Ech_con	Reniform echinate conical	Arecaceae
26	Ren_Ech_reg	Reniform echinate with regularly arranged projections	Arecaceae
27	Ren_Ech_clt	Reniform echinate with projections arranged in clusters	Arecaceae
28	Ren_Ech_wit	Reniform echinate with projections mainly on top	Arecaceae
29	L_Sph_Acu	Large spheroid phytoliths with acute projections	Arecaceae/Zingiberaceae
30	Sph_Tub	Spheroid tuberculate phytoliths	Arecaceae/Zingiberaceae
31	Sph_rd	Spheroid phytoliths with rounded projections	Arecaceae/Zingiberaceae
32	Sph_Ech_Reg	Spheroid echinate with regularly arranged projections	Arecaceae/Zingiberaceae
33	Sph_Ech_Crd	Spheroid echinate with crowded projections	Arecaceae/Zingiberaceae
34	Lf_Con	Leaf cones phytoliths	Arecaceae/Zingiberaceae
35	Sph_fld_Zin	Spheroid folded_Zingiberaceae morphotype	Zingiberaceae
36	Sph_Ech_Irr	Spheroid echinate with irregularly arranged projections	Arecaceae/Zingiberaceae/Bromeliaceae
37	Seh_Ech_Shb	Spheroid echinate with short, bold projections	Arecaceae/Zingiberaceae/ Bromeliaceae
38	Sph_Lar	Spheroid large granulate	Arecaceae/Zingiberaceae/ Bromeliaceae
39	Seh_Ech_Elcl	Spheroid echinate elongate with clustered projections	Arecaceae/Zingiberaceae/Bromeliaceae
40	Vlc	Volcaniform	Musaceae
41	Hat_Mus	Hat-shaped Musaceae phytoliths	Musaceae
42	Tec_protu	Tectangular (or squarish) with protuberances	Musaceae
43	Rd_protu	Roundish phytoliths with protuberances	Musaceae
44	Crs	Cross	Poaceae
45	Sd_uni	Saddle uniform	Poaceae, Chloridoideae
46	Sd_long	Saddle long	Poaceae, Bambusoideae
47	Sd_tall	Saddle tall	Poaceae
48	Sd_plt	Saddle plateaued	Poaceae, *Phragmites australis*
49	Sd_clp	Saddle collapsed	Poaceae, *Dendrocalamus* sp.
50	Sd_dbl	Double saddles	Poaceae, Chloridoideae
51	Pap_Sha	Papillate (nipple-like shaped)	Poaceae
52	Cre	Crenate	Poaceae
53	Rd_1	Rondel, morphotype 1	Poaceae
54	Rd_2	Rondel, morphotype 2 (*Oryza* sp.)	Poaceae, *Oryza sativa*
55	Rd_3	Rondel, morphotype 3 (*Zizania* sp.)	Poaceae, *Zizania* sp.
56	Bil_sym	Bilobate symmetric	Poaceae
57	Bil_asym	Bilobate asymmetric	Poaceae, Bambusoideae/Oryzeae/Panicoideae
58	Bil_tall_n	Tall narrow bilobate	Poaceae, Bambusoideae/Oryzeae/Panicoideae
59	Pol_1	Polylobate, morphotype 1	Poaceae
60	Pol_2	Polylobate, morphotype 2	Poaceae
61	Pol_3	Polylobate, morphotype 3	Poaceae
62	Trpz	Trapezoid varia	Poaceae
63	Bul_var	Bulliform varia	Poaceae
64	Bil_narr	Narrow bilobate	Poaceae, *Oryza sativa*
65	Bil_flab	Bulliform flabellate	Poaceae, Poaceae/Cyperaceae
66	Bdl_peak	Double-peak phytoliths	Poaceae, *Oryza sativa*
67	Sph_rug_lg	Large rugose spheroid	*Canna indica*
68	Spks	Spikes	*Cannabis* complex
69	Oval	Oval	*Cannabis* complex
70	Seg_Ova	Segmented ovals	*Cannabis* complex
71	Seg_Shpd	Club-shaped	*Cannabis* complex
72	Shapls	Shapeless	*Cannabis* complex
73	Abb_stl	Abbreviated stellate	Woody plants
74	Pol_ata	Polygonal plate	Woody plants; cf. *Vitex* sp.
75	Plt_elg	Plate-elongate	Woody plants
76	Rctg	Rectangle	Woody plants
77	Blo_wood	Woody blocky	Woody plants
78	Hair	Hair-like cells	Woody plants
79	Cf_cun	Cuneiform-like phytoliths of cf. *Azadirachta indica*	Cf. *Azadirachta indica*
80	(Poly)	Polyhedral (scalloped)	Cucurbitaceae
81	Bul_flab	Bulliform flabellate_Poaceae/Cyperaceae morphotype	Poaceae and Cyperacea; *Oryza sativa*
82	Sph_Dipt	Decorated spheroids of Dipterocarpaceae	Dipterocarpaceae (*Hopea* sp./*Shorea* sp.)
83	Poly_Mlt_Lg	large multifaceted polyhedrals	Annonaceae; *Zingiber* sp./*Curcuma* sp.
84	Sph_fld_Mango	Spheroid folded_*Mangifera indica* morphotype	*Mangifera indica*
85	Clp_hem	Hemispherical clump	cf. *Ligustrum* sp.
86	Strial_Terema	Pitted, striated phytoliths	*Trema* cf. (*orientalis*)
87	Ov_lg	relatively large decorated ovoids of 10-12 µm	Zingiberaceae, *Zingiber* sp.
88	Elg_smth	smooth-elongate	Zingiberaceae
89	Pola_plate	polygonal plate	Zingiberaceae or cf. *Vitex* sp.
90	Lg_point	long point	Zingiberaceae
91	Blo_var	Blocky varia	Various, indeterminate
92	Elo_ent_1	Elongate entire, morphotype 1	Various, Poaceae mainly
93	Elo_ent_2	Elongate entire, morphotype 2	Various, Poaceae mainly
94	Elo_Sin	Elongate sinuate	Various, indeterminate
95	Elo_det	Elongate dentate	Various, indeterminate
96	Elo_dend	Elongate dendritic	Various, indeterminate
97	Acu_Bul	Acute bulbose	Various, indeterminate
98	Sph_rug	Rugose spheroid	Various, indeterminate
99	Sph_orn	Ornate spheroid	Various, indeterminate
100	Ell_Ech	Ellipsoidal echinate asymmetric phytoliths	Various, indeterminate
101	Ell_grn	Ellipsoidal granulate	Various, indeterminate
102	Irr	Irregular sinuate	Various, indeterminate
103	Stel	Stellate	Various, cf. *Vitex* sp.
104	Plt_var	Plate-elongate	Various, cf. *Vitex* sp.
105	Rectg	Rectangle	Various, cf. *Vitex* sp.
106	Con_var	Conical varia	Various, indeterminate
107	Tra_ann	Tracheary annulate/helical	Various, cf. *Zingiber* sp.
108	ND	Nodular	Various, indeterminate
109	Amb_brn	Amoeboid branchiate	Various, can be also ferns
110	Amrf	Amorphic phytoliths	Various, indeterminate
111	Drusses	Other inorganic crystals and druses	Various, indeterminate

Notes: “morphotype” used together with phytolith description in cases where identification is uncertain but phytolith morphology suggests a certain phytolith type; if several types of phytoliths with the slightly different morphology is observed, numeration of types is applied: “morphotype 1”, “morphotype 2” etc.; cf. used together with plant names indicates possible source plants for this certain phytolith type.

If more than one possible assignment of the phytolith morphological type (morphotype) to the source plant(s) was possible, all plant groups were indicated, e.g., Arecaceae/Zingiberaceae/Bromeliaceae, Poaceae/Cyperaceae. Phytoliths for which more than three probable source plant groups were suggested after detailed morpho-comparative analysis and phytoliths occurring within a large number of plants were assigned to the ‘non-diagnostic phytoliths’. In case of doubt, when for any reason it was impossible to verify morphological assignment (only one phytolith of a certain type, poor preservation, ambiguous appearance) or if it was overall difficult to reveal a source plant, a conservative approach was applied and phytoliths with ambiguous morphology were grouped together with non-diagnostic phytoliths. If the preservation conditions and/or visual appearance of a phytolith did not allow for clear diagnostics of the phytolith (e.g., due to high level of erosion or because it appears largely broken), this was assigned to the group of ‘indeterminate phytoliths’. In all samples of the unprocessed palm leaves (i.e., fresh, dry and herbarized ones), phytoliths whose morphology and preservation allowed clear assignment to any taxonomical plant group, other than Coryphoideae palms, were treated as contaminants, i.e., material not originating from the studied palm-leaf material.

Phytolith identification was performed at the Department of Palynology and Climate Dynamics, University of Göttingen, and partly at the Institute for Wood Science, University of Hamburg. Microphotographs and microslides are kept at the Department of Palynology and Climate Dynamics, University of Göttingen and are available on request from the first author. Plant taxonomy of the phytolith source material followed the guidance of the Angiosperm Phylogeny Group (APG 2016).

Scanning Electron Microscopy (SEM) was applied in order to perform proper identification of the phytoliths registered in the palm leaf material and PLM samples. Small part of leaf tissue of herbarium specimen or a manuscript fragment was cut with an extra-sharp razor blade. The samples were coated in gold dust (BIO-RAD SEM Coating System) and examined in high vacuum by the field emission scanning electron microscope (FE-SEM) Hitachi S520 and FE SEM Quanta FEC 250 at the Institute for Wood Science, University of Hamburg.

### 2.5 Phytolith diagrams

Phytolith diagrams were prepared with TILIA/TILIA.GRAPH software (Grimm, 2004). Groups of samples with statistically similar phytolith assemblages were represented as local zones in the diagrams and were established based on the number-constrained cluster analysis by sum-of-squares implemented with CONISS for TILIA (Grimm, 1987). The stability of the classification and the sharpness of the clusters (probability, P; 1 ≤ P > 0) were tested using the bootstrap resembling performed in MULTIV (Pillar and Orlóci, 1996; Pillar, 1999).

Phytolith diagrams compare the phytolith assemblages from the analyzed samples and present relative abundances (percentages) of individual phytolith types, which were calculated on basis of the total phytolith sum (TPS) excluding malformed, eroded and largely broken phytoliths (indeterminate ones), silica sand and silicate lenses of unknown nature. Functional group sums as well as numbers of phytolith types identified within each group accompany percentage profiles. Furthermore, phytolith concentrations and contents are given. Full phytolith diagrams for palm leaf samples of *Borassus flabellifer* and *Corypha umbraculifera* can be seen in **Supplementary Materials S4** and **S5**, respectively. Raw phytolith counts for material of both species are available in **Supplementary Material S3.**

### 2.6 Multivariate data analysis

Multivariate data analysis (ordination) was performed in CANOCO for Windows 5.10 (Ter Braak and Smilauer, 2018) on (1) all phytolith data in order to study the relationship of the samples to their source material (i.e., fresh, dry, herbarized and manuscript material), morphological types of phytoliths and (in case it is possible) with the phytolith’s source plants. (2) Ordination of the unprocessed samples (i.e., of the fresh, dry and herbarized samples) was run separately in order to study potential relationships between these samples with their source material as well as with each other. These two analyses were performed separately on the samples of *Borassus* and *Corypha*. In both cases and for both species, a detrended correspondence analysis (DCA, *sensu* Hill and Gauch., 1980) was first conducted to determine the length of the composition turnover gradient, which resulted having a value of 1.5 for all samples together and 0.9 for unprocessed palm leaf samples (i.e., all samples excluding manuscript material). Thus, a linear approach is feasible, and a principal component analysis (PCA) was performed. Under the total variance was understood the ratio λ1 + λ2/total variance, equivalent to R2 (Jongman et al., 1987). (3) Additionally, ordination on the manuscript samples was performed in order to test these samples for their possible relationship to the palm species used as their writing supports, in order to reveal relationship of the PLM material to the phytoliths and (possibly) to the phytolith’s source plants. Here, samples of *Borassus flabellifer* and samples of *Corypha umbraculifera* were taken together. In each ordination, all taxonomic data were standardized and logarithmically transformed. To decrease the effect of rare phytolith types, only types that were present in at least 2 samples and in one of those a percentage value of >2% was reached were included in the final analysis. To evaluate the significance of the PCA axes in every case, the null hypothesis was tested using the nonparametric Monte Carlo permutation test (Manly, 1992). Under the null hypothesis, it is expected that no relations exist between the variation in phytolith percentages and sample nature. Ordination stability was checked as already described above, in the MULTIV software (Pillar and Orlóci, 1996; Pillar, 1999).

## 3 Results

### 3.1 Comparison of fresh, dry and herbarized palm leaf samples

#### 3.1.1 General patterns

**Figure 2:**
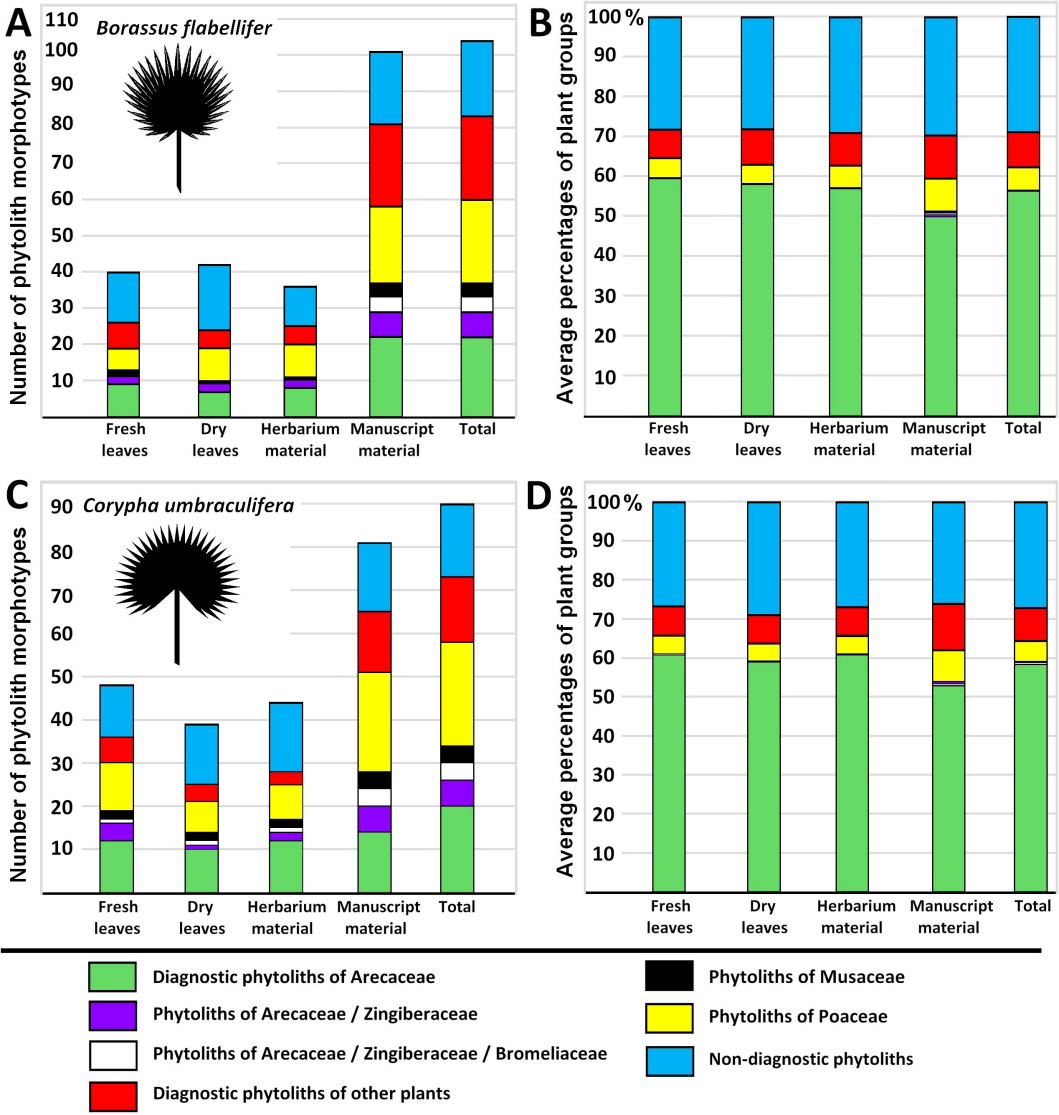
Numbers of identified phytolith morphotypes in **(A)** *Borassus flabellifer* and **(C)** *Corypha umbraculifera* material. Average percentages of main functional groups of plants identified based on phytolith assemblages in **(B)** *Borassus flabellifer* and **(D)** *Corypha umbraculifera* material.

We registered 111 unique phytolith types in total, 53 of these were only found in PLM samples. In *Borassus flabellifer* samples, 104 phytolith types were identified; and in *Corypha umbraculifera* 89 phytolith types were found (**Figure 2**). Arecaceae phytolith morphotypes included spheroid psilate, spheroid echinate and echinate symmetrical types, spheroid verrucate symmetrical and spheroid verrucate asymmetrical types, spheroid morphotype with acute projections, spheroid echinate morphotype with small, reduced, or undeveloped spines, spheroid echinate favose type, and aspherical echinate morphotype with sharp-rounded and roundish-triangulate spines, ranging from 9.5 to 14.5 µm in diameter. Both articulated and isolated spheroid phytoliths were grouped here. A complete list of phytolith morphotypes described from all study material and for both palm species is available in **Supplementary Material S3**.

**Figure 3:**
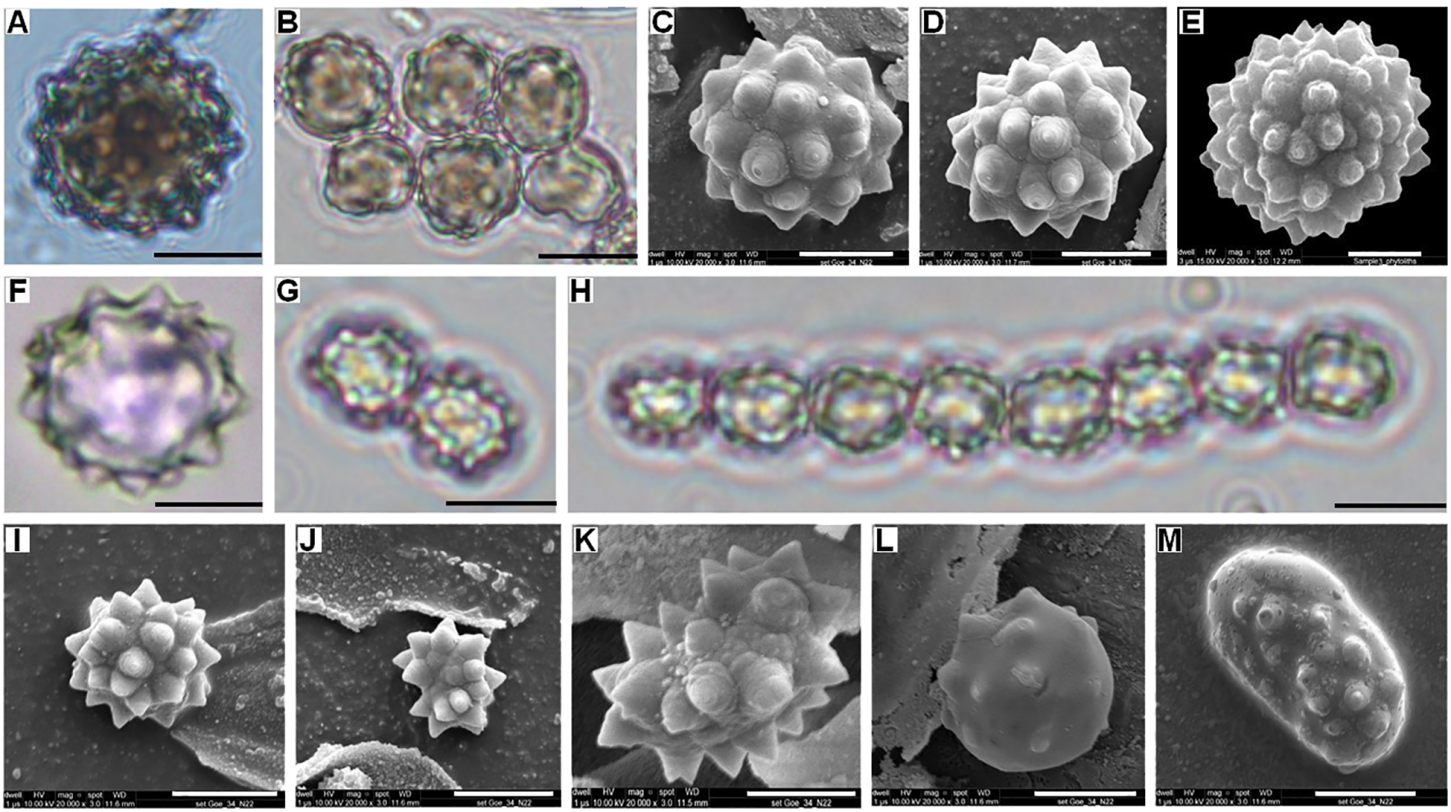
Palm phytoliths. Bright-field light microscope images of the **(A)** Isolated spheroid echinate body from *Borassus flabellifer* leaf; **(B)** Clustered spheroid echinate bodies from *Borassus flabellifer* leaf; FE-SEM pictures of **(C, D)** Spheroid echinate bodies from *Borassus flabellifer* leaf; **(E)** Large (>22 µm) spheroid echinate body with increased number of processes from cf. *Borassus flabellifer* inflorescence. Bright-field light microscope images of the **(F)** Isolated spheroid echinate body frzom *Corypha umbraculifera* leaf; **(G, H)** Clustered spheroid echinate bodies from *Corypha umbraculifera* leaf; FE-SEM picture of spheroid echinate bodies **(I)** From *Corypha umbraculifera* leaf; **(J)** Cf. Arecoideae phytolith from the surface of *Corypha* manuscript; **(K)** Reniform phytolith from the surface of *Corypha* manuscript; **(L)** Spheroid phytolith with mechanically brushed-off processes from the surface of *Corypha* manuscript; **(M)** Ellipsoid phytolith with mechanically brushed-off processes from the surface of *Borassus* manuscript. Scales of the micro-photographs are: for the light microscope images – 10 µm; for SEM images – 5µm.

In the unprocessed *Borassus* leaves 40, 42 and 36 types were registered for fresh, dry and herbarized material, respectively. In PLM samples of *Borassus*, 101 phytolith morphotypes were encountered, of which 23 were registered only in the manuscript material (**Figure 2A**). Phytolith assemblages of unprocessed *Borassus* leaves were dominated (ca 60% of TPS) by spheroidal echinate bodies of 15-20 µm (**Figures 3A–D**), thus, in the PLM material their contribution to TPS is reduced to ca 50% (**Figure 2B**). Unprocessed *Corypha* material yielded 48 types in fresh, 39 types in dry and 44 types in herbarized leaves. In the manuscript samples of *Corypha*, 81 phytolith types were found; 7 types are only registered in the *Corypha* manuscript samples (**Figure 2C**). spheroidal echinate bodies of 15-20 µm as well as smaller spheroid echinates (<7 µm) were constantly present in all samples (contributing from 59% to 61% altogether to the TPS, **Figure 2D**), but were absent from the record of *Corypha* PLMs. SEM microscopy clearly demonstrated that the small spheroids were a part of the *Corypha* leaf parenchyma. In addition, on the surface of PLMs of *Corypha*, spheroidal echinate bodies of a slightly different than Coriphoideae morphology were observed (compare **Figures 3F–J, L**). This concerns the number of spines in those that ranged from 10 to 15 (in Coryphoideae number of spines ranges from 17 to 21), and the length of spines ranging between 1.1 µm and 1.2 µm (in Coryphoideae they appeared to be larger: 1.5 μm to 1.8 μm). Spine edges of this morphological subtype are mostly rounded and rarely triangular or spiny as it is demonstrated by *Borassus* and *Corypha* spheroids. Ellipsoidal phytoliths with the morphology and morphometry of Coryphoideae were described as well (**Figure 3M**), thus ellipsoids are encountered not that frequently as spheroids. Additionally, in samples of *Borassus* large (20-25 µm in diameter; **Figure 3E**) echinate spheroids were registered 1-2 times in each material type. The aspherical echinate morphotype was only once registered in the fresh leaf material of *Corypha*; that could be a malformed spheroid.

**Figure 4:**
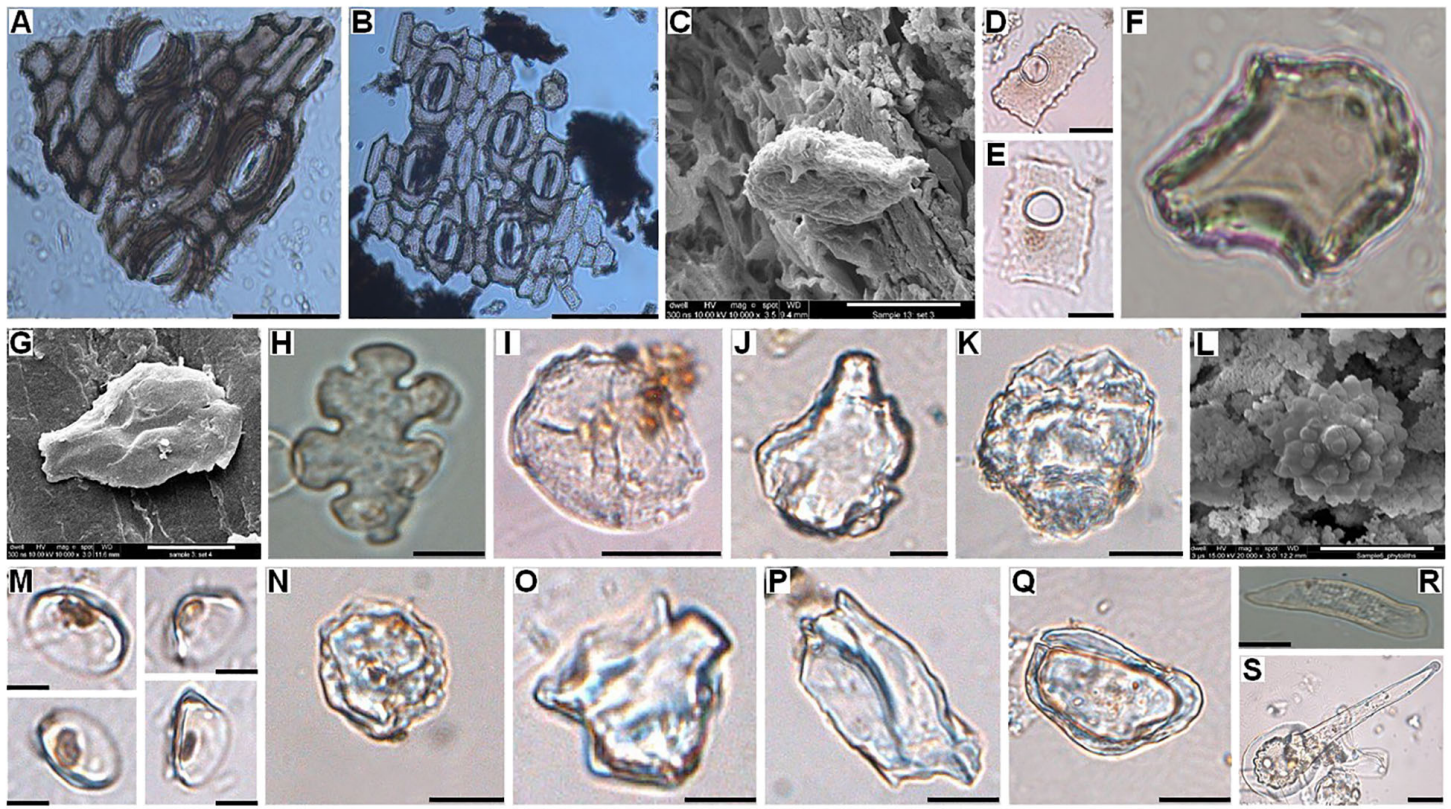
Bright-field light microscope images of **(A)** Epidermal tissue with stomata of *Borassus flabellifer* leaf material; **(B)** Epidermal tissue with stomata of *Corypha umbraculifera* leaf material; **(C)** FE-SEM picture of *Musa* sp. phytolith from the surface of *Corypha* manuscript; **(D, E)** Bright-field light microscope images of *Musa* sp. phytoliths from the surface of *Borassus* manuscript; **(F)** Bright-field light microscope image of the brown-colored burned bulliform flabellate phytolith of *Oryza* sp. from the surface of *Borassus* manuscript; **(G)** FE-SEM picture of bulliform flabellate phytolith of *Oryza* sp. from the surface of *Borassus* manuscript; Bright-field light microscope images of **(H)** Burned epidermal cell of *Mangifera indica* morphotype from the surface of *Borassus* manuscript; **(I)** Spheroid folded phytolith of cf. *Mangifera indica* morphotype from the surface of *Borassus* manuscript; **(J)** Bright-field light microscope image of cf. *Azadirachta indica* phytolith morphotype from the surface of *Corypha* manuscript; **(K)** Large decorated ovoid of Zingiberaceae plants from the surface of *Corypha* manuscript; **(L)** FE-SEM picture of spheroid echinate body from cf. Zingiberaceae/*Ananas comosus* from the surface of *Borassus* manuscript; Bright-field light microscope images of **(M)** *Canna indica* phytoliths from the surface of *Borassus* manuscript; **(N)** Mechanically destroyed pitted phytolith of cf. *Trema* (*orientalis*) from the surface of *Borassus* manuscript; **(O, P)** Dipterocarpaceae, cf. *Hopea* sp. phytolith morphotype from the surface of *Borassus* manuscript; **(Q)** Dipterocarpaceae, cf. *Shorea* sp. phytolith morphotype from the surface of *Borassus* manuscript; **(R)** Tracheary annulate/helical phytolith from the surface of *Borassus* manuscript; **(S)** Acute bulbose phytolith of unknown nature from the surface of *Borassus* manuscript. Scales of the micro-photographs are: for the light microscope **(A, B)** 20 µm; images **(D, E, M)** 5 µm; other images – 10 µm; for SEM images – 5µm.

Other common phytolith types in the unprocessed material of both species were isolated silicified stomata and stomata complexes (aggregated silicified stomata and leaf tissue cells; **Figures 4A, B**) of related species. Among the non-diagnostic phytoliths in both *Borassus* and *Corypha* leaves, various woody blocky and other blocky as well as hair-like and elongate entire phytoliths were observed (ca 30% of TPS in both species).

**Figure 5:**
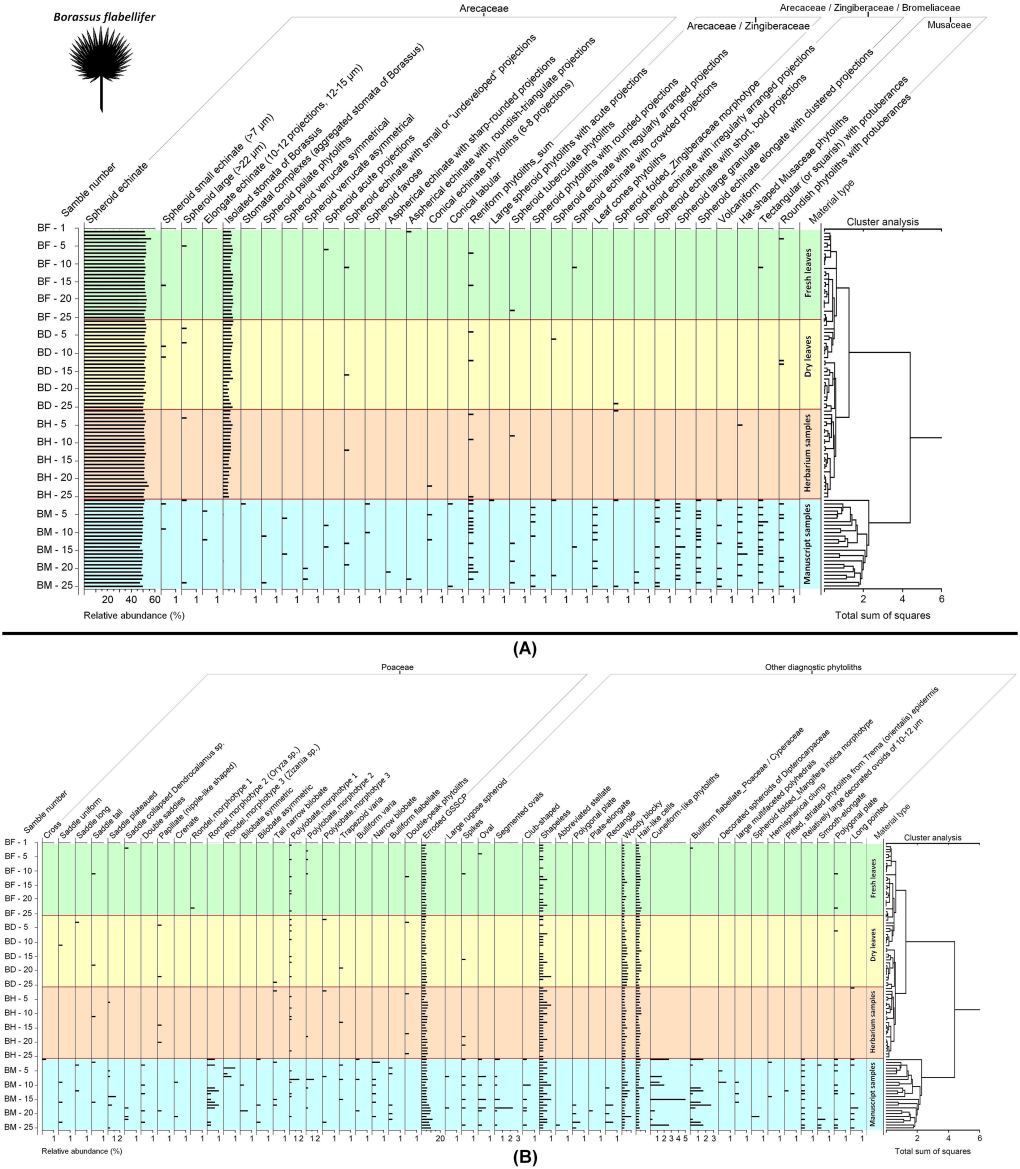
Diagram of phytolith analysis for fresh, dry, herbarized and manuscript samples of *Borassus flabellifer* showing relative abundances of the individual phytolith types within the groups of **(A)** Arecaceae, Arecaceae/Zingiberaceae, Arecaceae/Zingiberaceae/Bromeliaceae, and Musaceae phytoliths, **(B)** Poaceae and other diagnostic phytoliths. Functional groups of types are established based on their morphology and morphometry.

Results of phytolith analysis for both *Borassus* and *Corypha* with main parameters of the phytolith assemblage diversity, concentrations and contents of phytoliths in the analyzed 200 samples (100 samples of each species) are graphically presented in the **Figures 5**, **7** and summarized in **Table 3**. Reniform phytoliths (**Figure 3K**) are grouped in diagrams together. Complete diagrams showing all identified types, morphological varieties and druse-shaped inorganic crystals can be found in **Supplementary Material S4** for *Borassus* and in **Supplementary Material S5** for *Corypha*.

**Table 3:** Summary of the phytolith analysis run on fresh, dry, herbarized and PLM material of *Borassus flabellifer* and *Corypha umbraculifera*: percentages of TPS of functional phytolith groups are represented as well as some important types (i.e., phytoliths making *Cannabis* complex, phytoliths of *Oryza sativa*, phytoliths of cf. *Azadirachta indica*, phytoliths of *Dendrocalamus* sp., phytoliths of Zingiberaceae).

Type of material	BF	BD	BH	BM	CF	CD	CH	CM
Phytolith functional groups								
Arecaceae	60%	58% 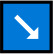	57% 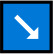	50% 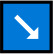	61%	59% 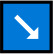	61% 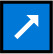	53% 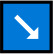
Arecaceae/Zingiberaceae	<1%	<1%	<1%	<1%	<1%	1% together	1% together	up to 1%
Arecaceae/Zingiberaceae/Bromeliaceae	no	no	no	<1%	<1%			up to 1%
Musaceae complex	no	no	no	up to 1%	<1%			<1%
Poaceae complex	5%	5%	5.5%	8% 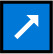	4%	4%	4%	8% 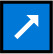 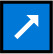
Other diagnostic phytoliths	7%	9% 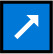	8% 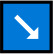	11% 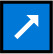	8%	7% 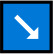	7%	12% 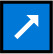 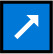
*Cannabis* complex	<1%	<1%	<1%	1% (up to 3%)	<1%	<1%	<1%	up to 1%
*Oryza sativa*	<1%	<1%	<1%	up to 1%	<1%	<1%	<1%	up to 1%
Cf. *Azadirachta indica*	no	no	no	<1%	no	<1%	<1%	3%
*Dendrocalamus* sp.	<1%	no	no	<1%	no	<1%	<1%	up to 1%
Zingiberaceae	<1%	<1%	<1%	up to 1%	no	no	no	no
Non-diagnostic phytoliths	28%	28%	29.5%	30% 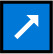	27%	29% 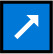	27% 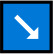	26%
Total number of registered types	40	42	36	101	48	39	44	81
Average number of registered types ± SD	13.2 ± 7.2	12.8 ± 3.7	13.4 ± 4.3	21 ± 8.3	15.5 ± 2.1	14.2 ± 1.4	13.7 ± 1.4	24.6 ± 4.8
Maximal number of registered types	26	24	24	41	20	18	17	35
Average phytolith concentration ± SD	27093 ± 439	4109 ± 436	4774 ± 188	4951 ± 272	24754 ± 461	25312 ± 309	26583 ± 289	20122 ± 1360
Average phytolith content ± SD	23186 ± 911	23224 ± 524	21903 ± 605	17045 ± 518	21826 ± 4702	21617 ± 3089	23667 ± 2879	17210 ± 1566

Main phytolith assemblage parameters (i.e., total number of registered types, average number of registered types, maximal number of registered types, average phytolith concentration, average phytolith content) are given with associated standard deviation (± SD). Dynamics of parameters (compare to the previous value of the same parameter) is demonstrated as following: increase - 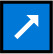 ; marked increase (five percent points and more)- 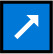 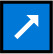 ; decrease - 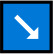. If none dynamics sign is shown, then no marked changes observed.

#### 3.1.2 Phytolith contamination of unprocessed palm-leaf material

Phytoliths from plants other than *Borassus* and *Corypha* were regularly found on the surface of fresh, dry, and herbarized palm leaves of these two species. As **Table 4** demonstrates, the most common phytolith contaminants of unprocessed leaves were GSSCP, which are small, lightweight, and prone to wind transportation. Virtually all of them (see **Tables 3**, **4**) were eroded, broken, and strongly degraded, with preservation notably different from the phytolith material collected from leaf tissues and PLM material. Presumably, most (if not all) of these degraded phytoliths originate from soil and/or dust.

**Table 4:** Phytolith contamination of the surface of fresh, dry and herbarized leaves (% of TPS).

	All Poaceae phytoliths	Eroded GSSCP	Other GSSCP	Eroded woody blocky	hear-like cells	Other
**BF**	4.46	4.40	0.49	2.60	4.15	1.76
**BD**	4.40	4.31	0.15	2.32	4.4	0.65
**BH**	4.39	4.28	0.18	2.60	4.28	0.90
**CF**	4.48	4.04	0.44	2.62	4.16	0.76
**CD**	4.32	4.19	0.13	2.37	4.4	0.55
**CH**	4.37	4.25	0.12	2.57	4.29	0.64

The total percentage of GSSCP contaminants in all analyzed unprocessed samples remained about the same (ca. 5% for both palm species; **Tables 3**, **4**; diagrams in **Figures 5**, **7**). In the manuscript samples, this amount was approximately twice as high (about 8% in both *Borassus* and *Corypha* samples). Other contaminants of unprocessed leaf material with distinctive origins (such as phytoliths of Musaceae or other than GSSCP phytoliths of Poaceae) occurred only randomly and rarely, i.e., 1-3 times per unprocessed material type in both palms. Generally speaking, fresh, dry, and herbarized leaves of *Borassus* appeared to be more contaminated than leaves of *Corypha* (compare the numbers of phytolith types per sample in *Borassus* vs. *Corypha* in **Figures 5** and **7**).

### 3.2 Phytoliths in PLM samples

In samples of PLMs, we observed all types of phytoliths described for fresh, dry, and herbarized material of the same species, although in different proportions (**Figures 2**, **5**, **7** and **Table 3**). Additionally, 68 other phytolith types were registered in PLMs that were never found in the unprocessed material. Among them, 46 types were registered for *Borassus* and 22 for *Corypha*. In *Borassus*, 23 unique types were observed (see **Figures 5**, **8**, and **Supplementary Material S3**), whereas 7 unique types were observed in *Corypha* (see **Figures 7**, **9**, and **Supplementary Material S3**). Manuscript samples were characterized by high variability in their phytolith assemblages; different manuscripts yielded different phytolith assemblages (**Figures 2B, D**, **5**, **7**). However, stomata and stomatal complexes, which were seen in each investigated sample of fresh, dry, and herbarized material, contributing 2% to 8% (**Figures 5**, **7**) to TPS, were not registered at all in the manuscript samples of either palm. In contrast, non-Arecaceae phytoliths were seen more often, in greater varieties and amounts in PLM samples. All these types were exclusively observed on the surface of the manuscript samples; some phytoliths (typically those of Poaceae and Musaceae) were forced into the palm-leaf tissue (**Figure 4C**), and many had a brown or blackish color (**Figures 4A, B, F, H**). Multivariate statistics revealed typical complexes of phytolith types occurring in the PLM samples.

**Figure 6:**
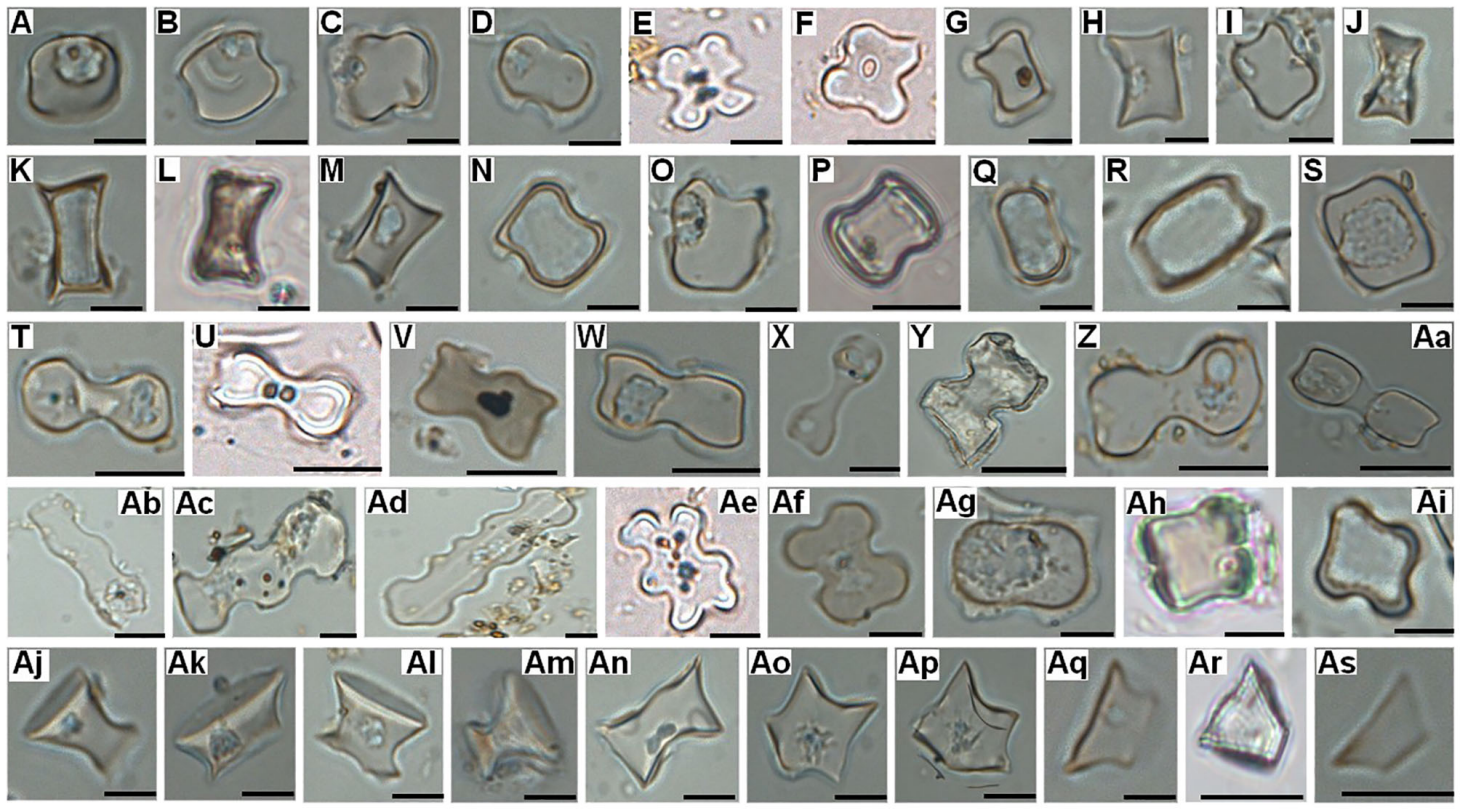
Grass silica short cell phytolith morphotypes (GSSCP) registered on the surface of various *Borassus* and *Corypha* PLMs. **(A-S)** Saddle; **(T-Aa)** Bilobate; **(Ab, Ac)** Polylobate; **(Ad)** Crenate; **(Ae-Ai)** Cross; **(Aj-Ap)** Rondel, **(Aq-As)** Trapezoid. Scales of the micro-photographs are – 10 µm.

**Figure 7:**
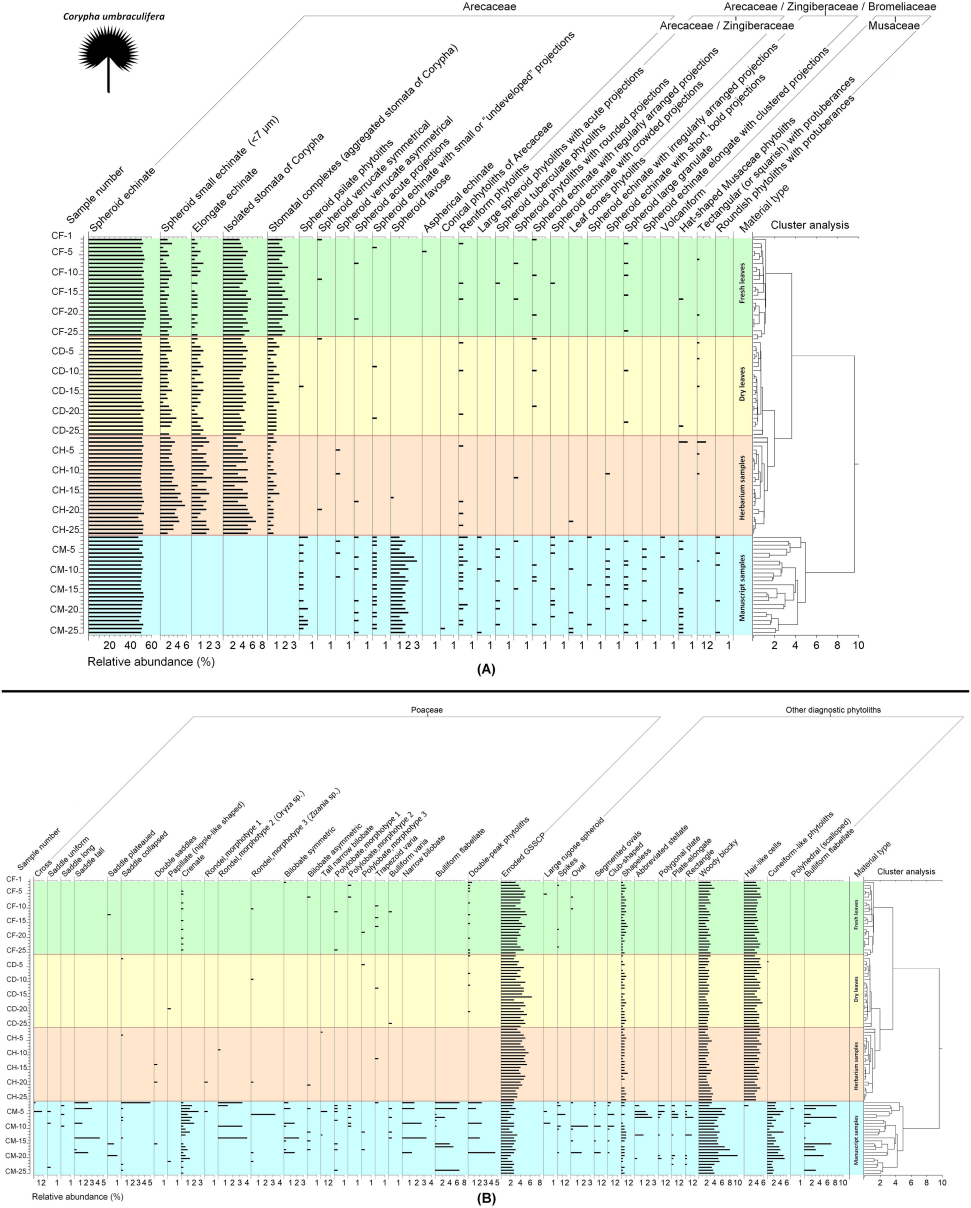
Diagram of phytolith analysis for fresh, dry, herbarized and manuscript samples of *Corypha umbraculifera* showing relative abundances of the individual phytolith types within the groups of **(A)** Arecaceae, Arecaceae/Zingiberaceae, Arecaceae/Zingiberaceae/Bromeliaceae, and Musaceae phytoliths, **(B)** Poaceae and other diagnostic phytoliths. Functional groups of types are established based on their morphology and morphometry.

**Figure 8:**
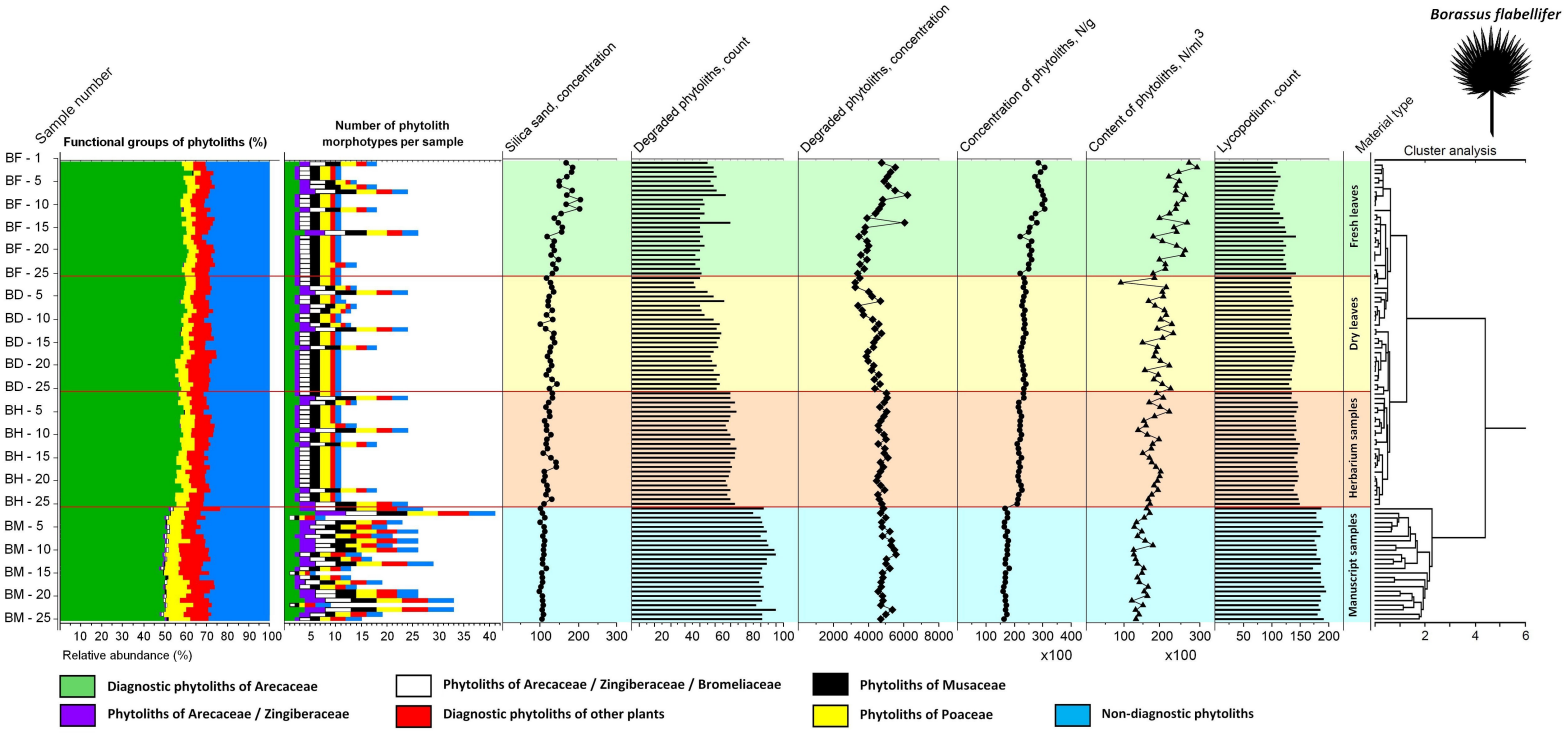
Summary diagram showing variability in the phytolith assemblages of the fresh, dry, herbarized and manuscript material of *Borassus flabellifer*, number of phytolith types registered, associated silica sand concentrations, degraded phytolith count and concentration, total phytolith concentration and contents as well as count on the *Lycopodium* markers used for their determination. Cluster analysis demonstrates similarities and dissimilarities in the analyzed samples.

**Figure 9:**
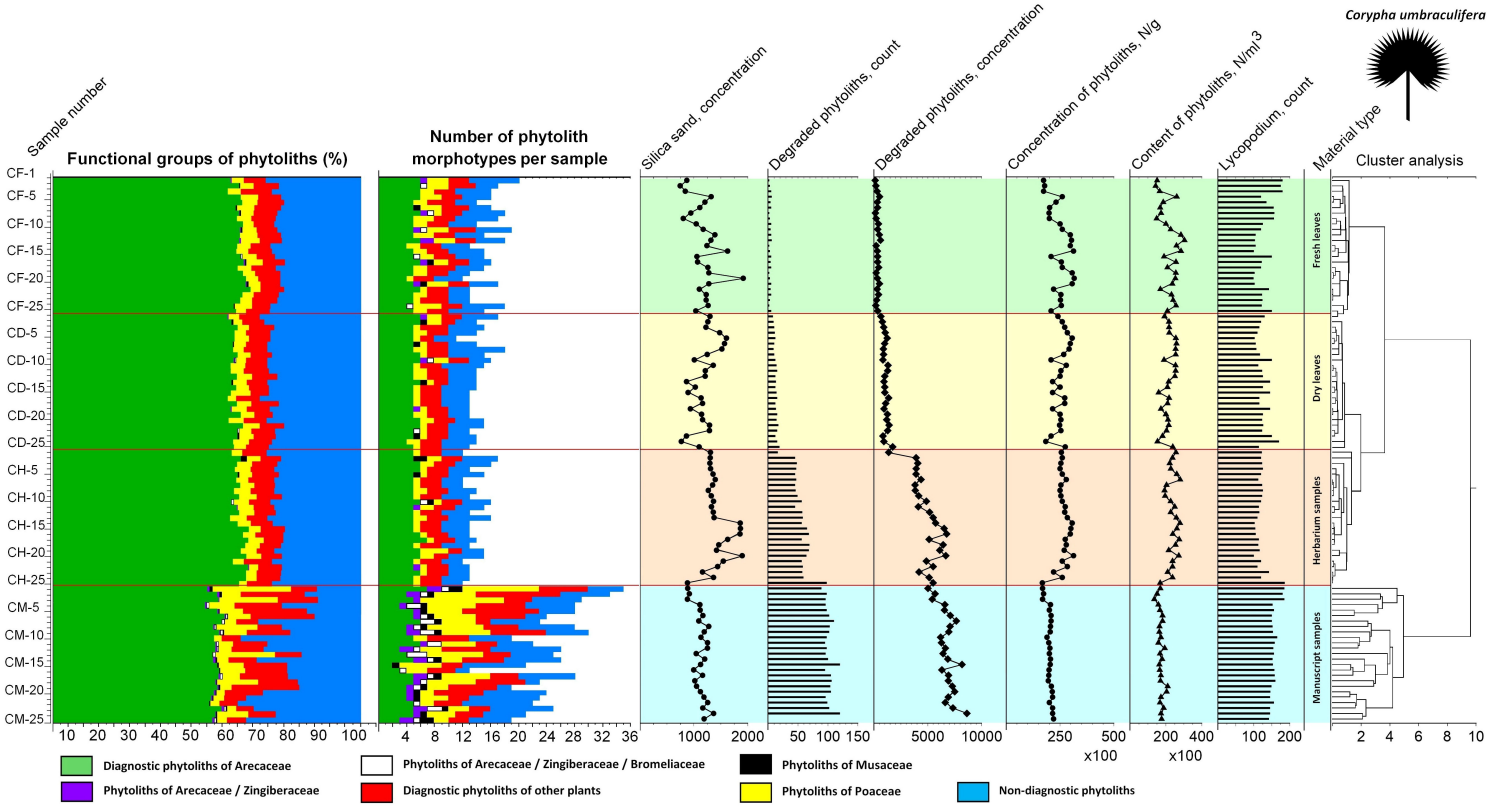
Summary diagram showing variability in the phytolith assemblages of the fresh, dry, herbarized and manuscript material of *Corypha umbraculifera*, number of phytolith types registered, associated silica sand concentrations, degraded phytolith count and concentration, total phytolith concentration and contents as well as count on the *Lycopodium* markers used for their determination. Cluster analysis demonstrates similarities and dissimilarities in the analyzed samples.

### 3.3 Results of multivariate analysis

#### 3.3.1 Cluster analyses: unprocessed and manuscript material are statistically separated

CONISS clustering (**Figures 5**–**8**) revealed distinct groups, with a probability of 0.74 for *Borassus* and 0.85 for *Corypha* palm leaves, separating PLM samples from unprocessed leaf samples. This was the first and most distinct cluster of samples in all diagrams. Fresh, dry, and herbarium samples were slightly better statistically separated for *Corypha* (0.61 probability, which is low), but still values of relative abundances of the phytoliths within all unprocessed samples make hardly a difference of 1%. For *Borassus*, fresh (BF-1 to BF-25) and the first 11 dry leaf samples (BD-1 to BF-11) were grouped together. Herbarium samples (BH-1 to BH-25) were grouped with the remaining 14 dry leaf samples (BD-12 to BF-25).

In the diagram for *Corypha*, the nature of all three types of unprocessed research material were revealed clearly and statistically significantly (within 2SD): clusters of FC (0.62 probability), DC (0.60 probability), and CH (0.64 probability) were derived. Clustering of the manuscript samples in both palm species was robust and statistically significant. The minimal probability for the separation of unprocessed and manuscript material was 0.59 for *Borassus* and 0.71 for *Corypha*. The null hypothesis that no relationships exist between the type of analyzed samples and relative abundance of the phytoliths within the samples was rejected for all *Corypha* samples at p=0.05. For *Borassus*, it was rejected for manuscript and unprocessed samples (p=0.05), but accepted for fresh/dry and dry/herbarium samples as these types of *Borassus* material failed to be distinguished by cluster analysis.

#### 3.3.2 Ordination: PCA analysis revealed three distinct phytolith complexes in PLM samples

**Figure 10:**
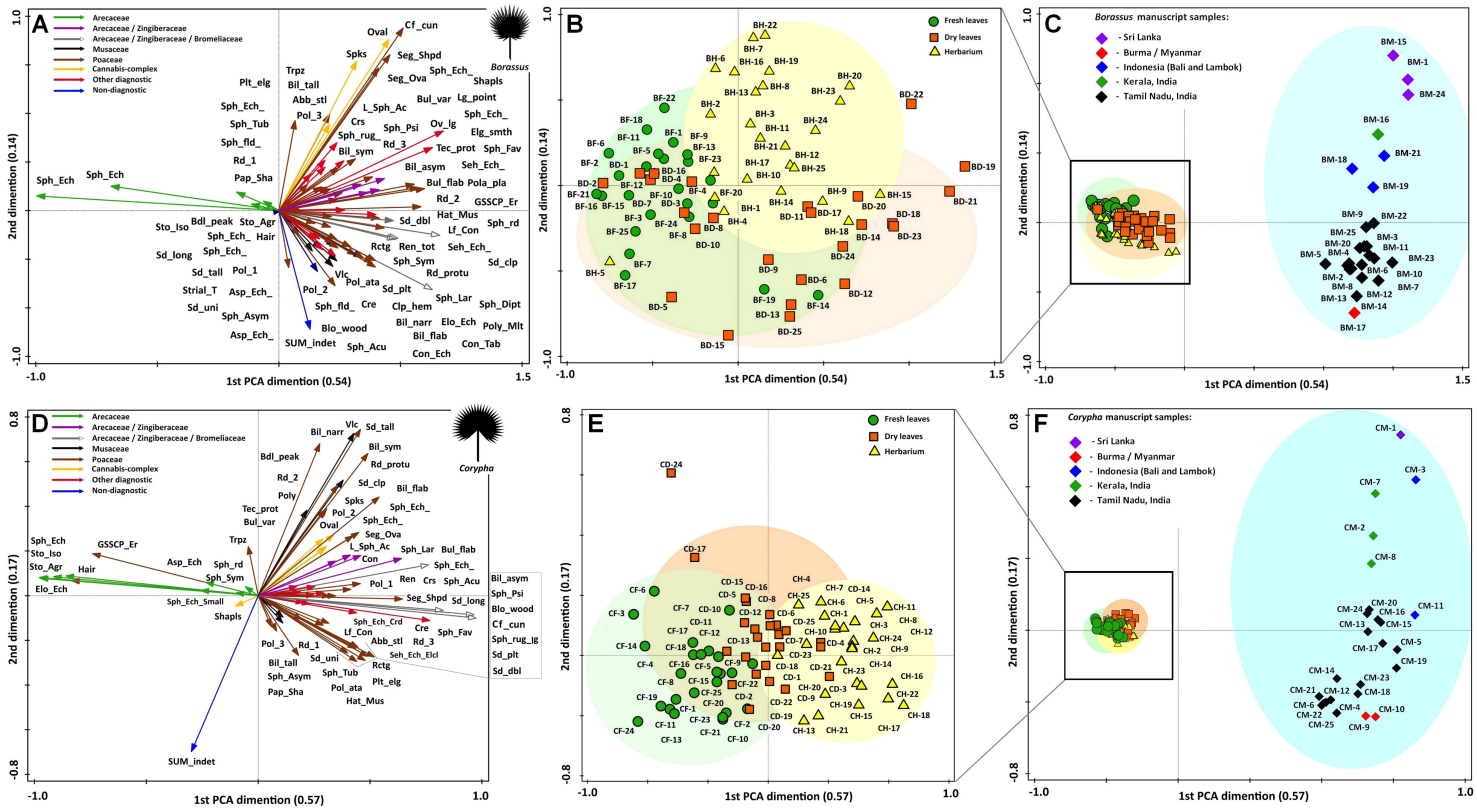
Results of principle component analysis (PCA) illustrating statistical relations of the most common and abundant phytolith types (shown with arrows) and samples collected from the indicated palm leave material of **(A-C)** *Borassus flabellifer* and *Corypha umbraculifera* **(D-F)**. The relative distance between samples explains the differences in phytolith composition. Groups of samples clearly reflect fresh (green clouds), dry (orange clouds), herbarized (yellow clouds) and manuscript (blue clouds) material. Ellipses are drawn at 0.95 of confidence. PCA on the fresh, dry and herbarized material for **(B)** *Borassus flabellifer* and **(E)** *Corypha umbraculifera* run separately.

Multivariate analysis performed on all 100 samples of *Borassus* demonstrated a measure of the goodness of fit equal to 0.87. The first two dimensions of the linear ordination (PCA, **Figures 10A–C**) account for 54% and 14% of the total variance of data. For all 100 samples of *Corypha* R2 = 0.89, and the first two dimensions of PCA (**Figures 10D–F**) taken 57% and 16% of the total data variance. For both palm species classification appeared rather sharp, with the minimal PCA clustering probability being 0.6 (for dry samples of *Borassus*). Otherwise, the PCA clusters probability varied from almost 0.9 (0.89 for manuscript samples of *Corypha* and 0.88 for manuscript samples of *Borassus*) to about 0.73 for other *Borassus* PCA clusters and about 0.86 for other *Corypha* PCA clusters, which is comparable to the results of the CONISS clustering described above.

Groups of samples of the unprocessed (BF, BD, BH) and manuscript material (BM) of *Borassus* are anticorrelated (mean r^2^ = - 0.61 ± 9.3 at p < 0.001; **Figure 10C**). For the unprocessed (CF, CD, CH) and manuscript (CM) samples of *Corypha* this anticorrelation is even stronger, with mean r^2^ = - 0.75 ± 5.3 (at p < 0.001; **Figure 10F**). The SD indices demonstrate, that the statistical variability of *Borassus* samples (taken together) is higher than of *Corypha*. That allows to conclude that in general *Corypha* samples are more homogeneous than *Borassus* samples in terms of their general phytoliths diversity and abundances. The same is also demonstrated by the direct count of phytolith types in the *Borassus* and *Corypha* material (compare **Figures 2A, C** and **8**, **9**).

For both species, strong correlation (mean r^2^ at p < 0.001 varies from 0.62 to 0.80) of the unprocessed material with spheroid echinate phytoliths, isolated silicified stomata and stomata complexes was revealed. Almost all non-Arecaceae phytoliths demonstrated either weak (from r^2^ = 0.3 to r^2^ = 0.01 at p < 0.001; 0 ≤ r^2^ < 1) correlation to the unprocessed samples of the both palms or an anticorrelation. Instead, non-Arecaceae phytoliths are strongly correlated with the PLM material (r^2^ = 0.51 to r^2^ = 0.9 at p < 0.001). Thus, eroded GSSCP and hair-like phytoliths appeared to be correlated positively with unprocessed samples of *Corypha*.

**Figure 11:**
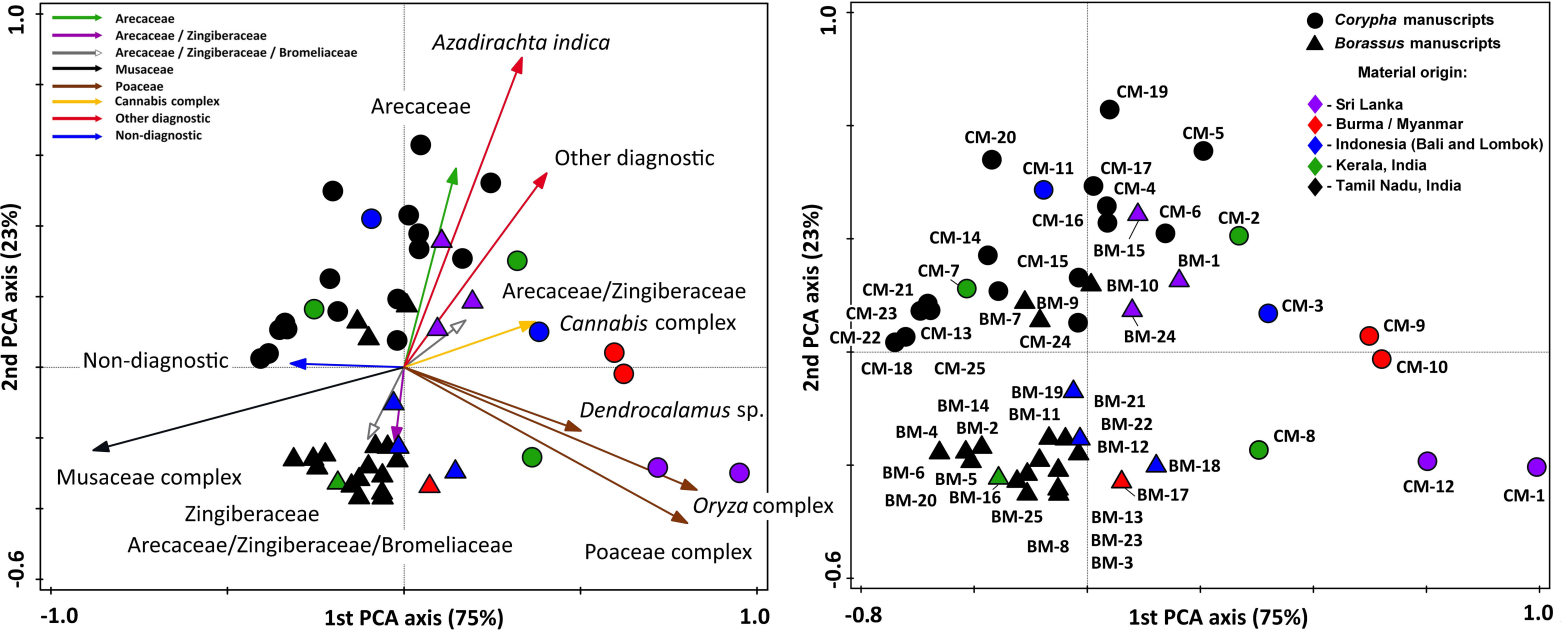
Results of principle component analysis (PCA) illustrating statistical relations of the manuscript samples and revealed phytolith source plants (shown with arrows). Samples of *Borassus flabellifer* (triangles) and *Corypha umbraculifera* (rounds) are analyzed together. The relative distance between samples explains the differences in phytolith composition. All registered phytoliths types are included into analysis, but only main functional groups and plants the most important for PLM creation are depicted on the graph.

It is worth mentioning that some spheroids were anticorrelated (r^2^ ≤ -0.6, p < 0.001; **Figures 10A, D**) with the unprocessed samples as well, i.e., spheroid tuberculate phytoliths, spheroid phytoliths with rounded projections, relatively large (18-20 µm) spheroid granulate phytoliths, isolated spheroid psilate phytoliths of a very small (1-1.5 µm) size (the last two are strongly correlated (r^2^ = 0.7 and higher at p < 0.001) with each other and with relatively large decorated ovoids of 10-12 µm in diameter, with smooth-elongate, tracheid, polygonal plate, and long point phytolith types. These phytolith complexes were observed in the same samples of *Borassus* manuscript samples (BM-3, BM-10, BM-12, BM-18, BM-20, and BM-21; **Figures 10A, C**, **11**).

The second distinct phytolith complex was composed of the spikes, ovals, segmented ovals, club-shaped and shapeless phytoliths (only in *Borassus*), those are very strongly (0.8 ≤ r^2^ < 1, p < 0.001) correlated to each other and occur in the same samples (BM-1, BM-7, BM-10, BM-15, BM-18 and CM-1, CM-5, CM-9, CM-18). In the samples of *Corypha*, shapeless phytoliths occur in both manuscript and unprocessed samples, thus demonstrating a weak correlation to any specific group of samples together with the non-diagnostic phytoliths (0.2 ≤ r^2^ < 1, p < 0.001; **Figures 10D, F**, **11**). The third complex of phytoliths was created by the rondels of morphotype 2, narrow bilobate and double-peaked bodies. They tended to occur together (as in the samples BM-1, BM-2, BM-3, BM-11, BM-13, BM-18 and in CM-1, CM-8, CM-13, CM-18; **Figures 10**, **11**) and were well correlated to each other (0.6 ≤ r^2^ < 1, p < 0.001). cuneiform bulliform phytoliths strongly correlated to the samples (CM-4, CM-5, CM-11, CM-17, CM-19, and BM-15; **Figures 10**, **11**).

Almost all saddle collapsed phytoliths in our record were dark-colored in our record. They were correlated with samples BM-18, BM-20, BM-22 and the majority (r^2^ = 0.64 at p < 0.001) also with CM-1, where this morphotype contributed 5% to its TOS, and that was the maximal for the saddle collapsed phytoliths in this record. Generally, material of both palm species demonstrated a high similarity in the phytolith assemblages among unprocessed samples (i.e., among samples of fresh, dry and herbarized leaves) and high dissimilarity among PLM samples.

Ordination performed on the manuscript samples of *Borassus* and *Corypha* taken together (**Figure 11**) reflected a low correlation (r^2^ = 0.34, p < 0.001) with a very low probability (P<50) indicating that this analysis failed to reflect any clear relationship of the analyzed samples to the palm species used as their writing supports. The samples are mixed, and the only significant group representing the biological nature of the palm leaves is the group of *Borassus* manuscript samples grouped in the lower-left part of the graph (**Figure 11**). These *Borassus* manuscript samples (BM-3, BM-5, BM-8, BM-11, BM-12, BM-13, BM-14, BM-16, BM-17, BM-18, BM-19, BM-21, BM-22, BM-23, BM-25) come from the same geographical area (Tamil Nadu, India; see **Table 1**) and were likely created using similar plants. Burned *Cannabis* sp. material characterized samples BM-1, BM-7, BM-15, CM-1, CM-5, and CM-18, and neem tree leaf material was evident (>3%) in CM-4, CM-5, CM-11, CM-16, CM-17, CM-18, CM-19, CM-20, and BM-15. All these manuscripts, except CM-11 (Balinese), are originally from Tamil Nadu, India, or Sri Lanka (see **Table 1**). In addition, we were able to determine larger (10-15 µm in diameter, **Figure 4M**) rugose spheroids of *Canna indica* (as described by Piperno and McMichael, 2020) on the surface of PLMs from BM-7 and CM-4 (Tamil Nadu, India), CM-8 (Kerala, India) and BM-18 (Bali, Indonesia).

## 4 Discussion

### 4.1 Phytolith assemblages and their potential source plants

As demonstrated by the results (**Figure 2**), phytoliths diversity in *Corypha* samples (89 morphotypes described in total) is less than the diversity of *Borassus* (104 of morphotypes), and the reason for that lays in the microstructure of the leaves surface of the compared palms. Leaves of *Corypha* are smoother and more tender, whereas *Borassus* leaves are uneven and rough on the surface, so they can collect much more ‘foreign’ micromaterial, i.e., phytoliths originating from non-Arecaceae plants. For this also speaks the phytolith morphology observed in the samples: types with more pronounced micro-sculptures on their surface, with processes, spines and ununiform bodies are preserved better and in grater abundance on the surface of *Borassus*, that is well seen e.g., in *Borassus* PLM samples. Each functional group of phytolith types described for fresh, dry, herbarized and manuscript material of both investigated palm species is discussed below.

#### 4.1.1 Arecaceae phytoliths

Most spheroid phytoliths of palms overlap in size, shape, spinule traits, and the number of surficial projections (Witteveen et al., 2022). For isolated (not aggregated) spheroid echinate phytoliths collected from PLM samples, we attempted to separate subfamilies of Coryphoideae and Arecoideae based on their morphometric characteristics. Arecoideae phytoliths, as described by Benvenuto et al. (2015), have a body size of 6-9 µm in diameter with a higher number of spines (10 to 15) compared to Coryphoideae (17 to 21). The length of spines for Arecoideae ranges between 1.1 and 1.2 µm (compared to 1.5 to 1.8 µm for Coryphoideae). Spine edges of this morphological subtype are mostly rounded. Ellipsoidal echinate phytoliths with comparable characteristics were also described, which we assumed could be a variety of the spheroid echinate phytoliths. Phytolith morphotypes of Arecoideae were never observed in the leaf tissue cuts of *Borassus* or *Corypha* palms, but only described from the surface of the PLMs and exclusively in an isolated form, suggesting they come from different Arecaceae species than those used as the writing support. Benvenuto et al. (2015) additionally described elongate psilate and elongate phytoliths with fusiform edges, as well as tabular sublobate phytoliths in Arecoideae, which we observed only seldom and did not assign to any specific plant group due to insufficient morphological evidence.

In addition to *Borassus* and *Corypha*, another species from Coryphoideae known for PLM production is *Phoenix dactylifera*, whose leaves are used as manuscript covers (Mahaparata, 1995). These leaves produce a large amount of spheroid echinate phytoliths (Bamford et al., 2006; described as globular echinate in the original publication), which comprise up to 67% of the phytoliths and do not vary much in size (Katz et al., 2010). Isolated spheroid echinate phytoliths described from PLM samples fit this description well, but it is impossible to discriminate them from other Coryphoideae phytoliths of a similar diameter (15-20 µm).

*Cocos nucifera* is used in PLM production for brushing material rubbed over the surface of the leaf (Bisoi, 1995) and its ash is presumably used for writing, inking, and/or making engraved script visible and more readable (Subramaniam, 1995; Harinarayana, 1995; Nishanthi and Wijayasundara, 2022). This could explain why some Arecaceae phytoliths identified from PLM samples show clear signs of contact with open fire (partially melted and blackish or brownish in appearance).

Additionally, Sharma et al. (2021) mention the use of coconut oil and palm kernel oil (produced from *Elaeis guineensis*), but oils are unlikely to be a source of palm phytoliths. The use of *Areca catechu* leaves for polishing manuscripts is indicated by Alahakoon (2012) and Subramaniam (1995). Fenwick et al. (2011) described *Areca catechu* and *C. nucifera* phytoliths as spherical to ellipsoidal, with body sizes of approximately 7.68 µm and 9.25 µm, respectively, which aligns with our observations of small Arecaceae phytoliths from PLM surfaces. Fenwick et al. (2011) also note that *C. nucifera* demonstrates a large percentage of reniform phytoliths (15.6% of their assemblage). We observed some reniform phytoliths (**Figure 3K**) on PLMs characterized by low amounts of Poaceae phytoliths. Considering the frequent use of the grass parts and products, namely straw, bran, and husks as cleaning and brushing material before writing a text (e.g., Sah, 2002; Meher, 2009; Alahakoon, 2012), we assume that in cases where reniform phytoliths are found on the PLM surface and few or no Poaceae phytoliths are present, *C. nucifera* leaves or fruit shell material were used for polishing.

Reniform phytoliths (**Figure 3K**) were rarely registered in the unprocessed samples (2-3 samples of each material type for *Borassus* and 3-4 times for *Corypha*), but their frequency increased about threefold in manuscript samples (15 in *Borassus* and 17 in *Corypha*). We assume these phytoliths do not originate from the palm tissues of the studied species, at least not all of them. Conical echinate and conical tabular phytoliths resemble those described by Witteveen et al. (2022) for *Bactris* sp. and *Bactris simplicifrons*, respectively. As *Bactris* are American palms and not native to India, and since Arecaceae conical phytoliths were only seen in herbarium material, it is highly likely that cf. *Bactris* contamination resulted from storing *Borassus* and *Bactris* collection folders together in the same cupboard.

#### 4.1.2 Arecaceae/Zingiberaceae phytoliths

In many cases, it is difficult or impossible to distinguish between Arecaceae and Zingiberaceae phytoliths without explicit knowledge of their exact plant sources (e.g., Tomlinson, 1990; Kealhofer and Piperno, 1998; Benvenuto et al., 2015). Therefore, phytoliths with overlapping morphology, such as large spheroid phytoliths with acute projections (up to 12 µm), spheroid tuberculate phytoliths, spheroid phytoliths with rounded projections, spheroid echinate silica bodies with regularly arranged projections, and spheroid echinate phytoliths with crowded projections and leaf cone phytoliths, were assigned to both families inseparably. Benvenuto et al. (2015) indicate that not only qualitative morphological similarities but also quantitative characters like length and height show strong overlap in phytoliths of species from Marantaceae and Orchidaceae, complicating family differentiation. Other authors note that sphere and spheroid phytoliths with irregular surfaces are seen in Anacardiaceae (Kealhofer and Piperno, 1998), Marantaceae, and Zingiberaceae (Brilhante de Albuquerque et al., 2013). The use of Anacardiaceae in PLM production is unknown. While Marantaceae is common in the New World (Andersson, 1981; Brilhante de Albuquerque et al., 2013), in SE Asia, only *Phrynium* and *Cucurligo* are known from northern Thailand forests (Kealhofer and Piperno, 1998).

Orchidaceae phytoliths can also be easily confused with those of Arecaceae and Zingiberaceae (e.g., Kealhofer and Piperno, 1998) as their spheroid phytoliths have comparable size (4-14 µm) and undistinguishable morphology. The presence of Orchidaceae phytolith material in the PLM samples remains unclear. So far, no mention of these plants in PLM production literature was found (Poliakova et al., in preparation). While their ethnobotanical significance could imply usage, more research is needed to confirm this. Benvenuto et al. (2015) note the difficulty in separating Zingiberaceae phytoliths because many genera produce no silica or only small, indistinct phytoliths. We regularly observed small (1-1.5 µm) isolated spheroid psilate phytoliths, but their classification was challenging. Chen and Smith (2013) indicate that Zingiberaceae seed phytoliths do not differ from other vegetative material, though folded, decorated spheres in Zingiberaceae are noted (Kealhofer and Piperno, 1998). Similar morphotypes are observed also in Anacardiaceae that could be the alternative source for these phytoliths in PLM material.

Large multifaceted polyhedrals are known in *Zingiber* sp. and *Curcuma* sp (Kealhofer and Piperno, 1998), and both plants are important in PLM production. *Zingiber officinale* is used as a conserving agent (Sah, 2006; Sahoo and Mohanty, 2007), and turmeric (*Curcuma longa*) is applied for leaf seasoning (Chakravarti, 1897; Sahoo and Mohanty, 2004; Sah, 2006), coloring with turmeric juice (Wilson and Rice, 2019), and protection (Bisoi, 1995; Padmakumar and Sreekumar, 2003). Multifaceted polyhedrals of *Zingiber/Curcuma* were observed in all PLM samples from India and Sri Lanka. We also found large decorated ovoids (up to 10-12 µm; **Figure 4K**) of *Zingiber* sp. described by Kealhofer and Piperno (1998), particularly in Sri Lanka manuscripts. Wang et al. (2022) identify smooth-elongate, tracheid, polygonal plate, and long point phytolith types in Zingiberaceae in subtropical Southwest China. These types were described in our PLM samples, supporting the literature that Zingiberaceae were actively used in PLM production. However, these indicators are insufficient to specify particular plant genera or species.

#### 4.1.3 Arecaceae/Zingiberaceae/Bromeliaceae phytoliths

As discussed by Chen and Smith (2013) and Benvenuto et al. (2015), spheroid echinate phytoliths with irregularly arranged projections, spheroid echinate with short and bold projections, large granulate spheroid phytoliths, and spheroid echinate elongate with clustered projections are difficult to attribute to any specific family. Besides Arecaceae and Zingiberaceae, these phytolith types are also described from Bromeliaceae, Strelitziaceae (Tomlinson, 1990; Kealhofer and Piperno, 1998; Benvenuto et al., 2015), as well as Cannaceae (Pearsall and Dinan, 1992; Kealhofer and Piperno, 1998; Brilhante de Albuquerque et al., 2013), and some Cyperaceae (Wallis, 2003). However, we have no clear evidence of all these families being used for PLM production. Additionally, plants from these families (except Zingiberaceae and Bromeliaceae) are not mentioned in the analyzed literature (Poliakova et al., in preparation).

Regarding Bromeliaceae specifically, this plant family is indigenous to South America; there are no bromeliads native to S and SE Asia (Mabberley, 1997; Gouda et al., 2022). The only Bromeliaceae plant historically known in S and SE Asia is the pineapple (*Ananas comosus* (L.) Merr.), introduced by the Portuguese in 1548 CE. Therefore, all possible identifications of Bromeliaceae phytoliths have to be attributed to *Ananas*. According to a study by Corrales-Ureña et al. (2018), the bracts and shell of the pineapple consist of spheroid echinate phytoliths 5 to 10 μm in diameter (described as rosette-like silica-based microparticles with an average size of 8.4 ± 2.5 μm, formed by even smaller micro- and nanoparticles). These are morphologically very similar to those described from palms (compare **Figures 3C, D** with **Figure 4L**). Similar phytoliths were also observed in pineapple by Ferreira and de Araujo (2010). Sah (2002) mentions the use of pineapple in Sinhalese PLM practices, noting that leaves (not fruits) were used. We extracted isolated phytoliths of this morphotype from inside the palm leaf tissues of fresh and dry material and additionally described them from the surface of the PLMs as isolated silica bodies. Considering their similar appearance, overlapping morphometrical parameters, and without knowing the exact origin and taxonomic source of these phytoliths, we cannot conclusively determine their exact source. Therefore, we maintain our identification at the level of the family group.

#### 4.1.4 Musaceae phytoliths

Volcaniform phytoliths (also described as vegetative trough morphotypes; **Figures 4C–E**) of diverse varieties, hat-shaped phytoliths, rectangular (or squarish) phytoliths with protuberances, and roundish phytoliths with protuberances were identified as banana phytoliths, as proposed by Mbida Mindzie et al. (2001); Ball et al. (2006); Neumann and Hildebrand (2009), and Chen and Smith (2013). The presence or absence of processes and the arrangement of ridges in tabular seed phytoliths have been suggested as diagnostic features to distinguish between the genera *Ensete* and *Musa* (Lentfer, 2009; Perrier et al., 2011). However, due to the limited reference phytolith material available and the state of preservation not always allowing for proper description of the surface texture of the cone and basal parts of the phytoliths, we did not distinguish troughs based on their fine morphology at this pilot phase to avoid overinterpretation. Based on the micro-characteristics of the eight types of volcaniform phytolith morphotypes proposed by Vrydaghs et al. (2009), we attempted to identify phytoliths of edible banana (*Musa acuminata* Colla) when the preservation state allowed it.

Certain hat-shaped phytoliths with thin bases are also produced by Marantaceae and Lowiaceae (Benvenuto et al., 2015), but they can be well discriminated from the hat-shaped phytoliths of Musaceae. Moreover, these phytoliths are prone to dissolution and may be underrepresented in phytolith assemblages (Benvenuto et al., 2015; own observations). Furthermore, no plants from Marantaceae and Lowiaceae families are known to be used for PLM production (Poliakova et al., in preparation), and we did not observe any similar types in our samples.

#### 4.1.5 Poaceae phytoliths

In the samples collected from PLMs (and exclusively therein), we observed all typical GSSCP (Twiss et al., 1969; Terrell and Wergin, 1981; ICPN 2.0, 2019; **Figure 6**). Papillate (nipple-shaped) silica bodies, presumably originating from grass bracts (Kaufman et al., 1972; Sangster et al., 1983; Piperno, 2006), culms, and leaves (Brown, 1984; Ball et al., 1993) were observed in manuscript samples from Kerala and Indonesia together with specific Chloridoideae saddles and double Chloridoideae saddles (Cordova, 2023).

Collapsed saddle and bamboo bulliform flabellate phytoliths (**Figures 4F, G**) observed in Indonesia samples, indicated as characteristic of *Dendrocalamus giganteus* and *Dendrocalamus peculiaris* (Wang et al., 2022), suggest that *Dendrocalamus* was probably also used at some stages of PLM production. Other evidence for bamboo (Bambusoideae) includes long saddles (Gu et al., 2016; Li et al., 2014; Tao et al., 2019), observed in some Indian samples. Most of these silica bodies are dark-colored and sometimes melting-deformed at the edges, suggesting possible burning of this particular plant material, likely if *Dendrocalamus* sp. was used for making and supporting a fire (Elbaum et al., 2003; Parr, 2006). Bamboo stems are a common and inexpensive fire fuel in S and SE Asia. For CM-1, *Dendrocalamus* sp. phytoliths appeared neither burned nor fire-deformed but mechanically broken, pressed, and/or driven into the manuscript surface. This suggests bamboo material in CM-1 was used for brushing or smoothing the manuscript folios.

The phytolith complex of tall narrow bilobate, saddle, tall saddle, and bilobate bodies observed in the same sample can indicate the use of Bambusoideae, Oryzeae, or Panicoideae grasses (Kealhofer and Piperno, 1998; Dai et al., 2023). However, we suggest these assemblages are more indicative of Oryzeae, serving as proxies for rice, as these types were often found with considerable amounts (10-12% of the total phytolith sum) of bilobate, bulliform, and double-peak phytoliths diagnostic for domestic rice (Gu et al., 2013; Ma et al., 2016). We cannot, however, completely disregard other plant sources.

Determining the use of domestic rice (*Oryza sativa* L.) and wild rice (*Zizania* sp.) is culturally and botanically important. Yost and Blinnikov (2011) identified 23 different morphotypes in *Zizania palustris* L. locally diagnostic of the species in the USA (Minnesota). They indicated one morphotype potentially diagnostic of wild rice (inflorescence morphotype 1). Similar phytoliths were only found in manuscript material from Burma/Myanmar (BM-17, CM-9, and CM-10), along with rondel morphologies, suggesting the use of *Zizania* sp. rather than *Zea mays*, as maize is not mentioned for PLM production, whereas rice is regularly referred to (e.g., Sah, 2002; Padmakumar and Sreekumar, 2003). Although we did not find diagnostic phytoliths of *Zea mays* (e.g., cross phytoliths larger than 21 μm or wavy-top rondels; Iriarte, 2003; Piperno, 2006; Witteveen et al., 2024), the absence of such phytoliths aligns with our assumption concerning rice usage.

Plateaued saddle morphotype suggests the use of *Phragmites australis* (Cordova, 2023). Elongate entire, i.e., flat rectangular epidermal cell phytoliths, are produced by all grasses (ICPN 2.0, 2019), supporting the hypothesis of active Poaceae use in PLM creation. However, bulliform flabellate phytoliths occur in both Poaceae and Cyperaceae (Esau, 1965; Metcalfe, 1960, Metcalfe, 1971), making it difficult to use them as definitive diagnostic types for grasses. These phytoliths, although present in PLM samples and absent in fresh, dry, or herbarium material, suggest possible use of Poaceae in combination with Cyperaceae at some stages (e.g., burring, boiling, and/or polishing) of PLM production.

#### 4.1.6 Phytoliths diagnostic for other plants

Phytolith analysis demonstrates that samples of PLM from Tamil Nadu (India, see **Table 1**) were actively treated with *Zingiber/Curcuma* (Zingiberaceae) and possibly with pineapple (the only Bromeliaceae in the study regions). Some of Indian manuscripts (See results) were treated with banana leaves (Musaceae) and/or *Canna indica*. One *Borassus* manuscript (BM-10), although grouped with *Corypha* samples in the last ordination run (**Figure 11**) due to the presence of *Cannabis* sp. and cf. *Azadirachta indica* phytoliths, was likely treated with the same plants as well. The last two plants were found in many *Corypha* manuscript samples (**Figures 7**, **9**) used for analysis.

Neem tree is a crucial plant in PLM production (Joshi, 1995; Perumal, 2013; Nishanthi and Wijayasundara, 2022). Phytoliths of cf. *Azadirachta indica* (described as ‘cuneiform bulliform’ by Gasma et al., 2022) were mainly found in the manuscript material of *Corypha* palm originating from India and Sri Lanka, aligning with the literature on PLM creation and conservation. Neem leaf and burned *Cannabis* sp. material was evidenced in Tamil, Sri Lanka and Balinese (only in CM-11) manuscripts, where the neem tree is often mentioned as a plant used in PLM production (e.g., Joshi, 1995; Sahoo and Mohanty, 2004; Sah, 2002; Nishanthi and Wijayasundara, 2022) and is still used as a manuscript conservation agent (Subramaniam, 1995) and a ritual plant (Arumugam, 2020). In some parts of Tamil Nadu, neem trees are worshiped as goddesses (Hertzman et al., 2023), and if the manuscript contain any holy text, they can be also treated with neem leaves for ritual purposes. *Cannabis* sp., an important ritual plant in India and surrounding countries, is evidenced in the PLM samples by the phytolith complex described by Golokhvast et al. (2018). This includes oval, segmented oval, club-shaped, spikes, and shapeless phytoliths (for *Borassus*), all 20-50 µm in diameter and 20-70 µm in length, as also seen in our samples (**Figures 5**, **7**–**9**, **11**). Almost all manuscript samples originating from India, especially BM-15 and BM-18 from Tamil Nadu, bear traces of the *Cannabis* plant on their surface.

*Vitex negundo*, used for the preparation and conservation of PLMs, is believed to protect the folios from rodents, insects, and mold (Joshi, 1995; Sah, 2006; Sahoo and Mohanty, 2007; Meher, 2009; Sharma, 2018; Wilson and Rice, 2019; Sharma et al., 2021). Common phytolith types for *Vitex* include abbreviated stellate, polygonal plate (which can also come from Zingiberaceae), plate-elongate, rectangle, woody-block, and hair-like cells (Wang et al., 2022). Although unequivocal identification based on this phytolith complex is not feasible, *Vitex* can be considered one of potential sources of the mentioned silica bodies on the PLM surfaces.

Microphotographs presented by Kealhofer and Piperno (1998) and our reference material allowed the identification of some decorated spheroids of Dipterocarpaceae (*Hopea* sp./*Shorea* sp. type; **Figures 4O–Q**), with distinct ornamentation found in *Borassus* manuscripts BM-5 and BM-9, as well as spheroid folded phytoliths of *Mangifera indica* morphotype (BM-21; **Figures 4H, I**) and pitted, striated phytoliths of *Trema* (*orientalis*) epidermis (BM-12; **Figure 4N**). However, these single findings do not allow to draw any conclusion on the use of these plants in PLM creation.

A few scalloped large (ca. 40 µm) roundish phytoliths of domesticated *Cucurbita* sp (Piperno et al., 2002; Piperno, 2006). appeared in two samples of Indian *Corypha* manuscripts. The *Cucurbita* sp. is not mentioned in PLM production literature. Furthermore, these well-preserved phytoliths were not covered by patina and cannot be considered part of the PLM production process. There is no possibility of confusing these phytoliths with those of *Lithocarpus* sp., as the latter forms distinctive, faceted, spheroidal polyhedrals (Kealhofer and Piperno, 1998), which are normally about half the size of Cucurbitaceae phytoliths. It is most likely a modern contamination.

### 4.2 Non-diagnostic phytoliths

In this group we aggregated those phytolith types that can occur in different non-related plants. We group here psilate spheroid phytoliths, produced by many monocots (Witteveen et al., 2024) and woody taxa such as *Syzygium aromaticum* (Dai et al., 2023), which is an important component of boiling solutions and used as an insecticide (Sah, 2002; Sahoo and Mohanty, 2004, Sahoo and Mohanty, 2007; Sharma et al., 2018, Sharma et al., 2021), as well as an oil source (Joshi, 1995) in PLM production.

Rugose spheroid phytoliths, produced most abundantly by Chrysobalanaceae, are notorious for confounding phytolith distinctions (ICPN 2.0, 2019). These are also produced by Lecythidaceae, Moraceae, Malvaceae, and Proteaceae (Piperno and McMichael, 2020). Ornate spheroid phytoliths can be produced by Acanthaceae, Burseraceae, Lecythidaceae, Malvaceae, Moraceae, Violaceae, Vochysiaceae (Piperno and McMichael, 2020), as well as by Dipteridaceae, Rubiaceae, and Rutaceae (Dai et al., 2023). However, it is challenging to determine the specific source plant group in our case.

Blocky phytoliths are common in Poaceae and Cyperaceae but are difficult to interpret without anatomical context. They are also found in various monocots, dicots (Tsartsidou et al., 2007; Collura and Neumann, 2017), and conifers (Strömberg, 2003). We described these from all types of studied material, and they do not hold significant taxonomic or ethnobotanical meaning for our study.

Elongate entire phytoliths are frequent in many plants, and their taxonomic diagnostic value is low (ICPN 2.0, 2019). Acute bulbose phytoliths (**Figure 4S**) are often described from grasses (e.g., Brown, 1984; Piperno, 2006; ICPN 2.0, 2019), sedges (Strömberg, 2003), certain dicots, palms, and occasionally from *Equisetum* and *Selaginella* (Strömberg, 2003). Since we frequently observed these types in fresh, dry, and herbarized palm leaf material, and not exclusively in PLM samples, we do not consider these phytoliths as indicators of other plants.

Elongate sinuate phytoliths and their transitional forms are suggested to be attributed to Poaceae when accompanied by other typical Poaceae morphotypes, such as acute bulbose, papillate, and graminaceous stomata (Metcalfe, 1960; ICPN 2.0, 2019). Considering the variety of potential phytolith sources in our study material (especially for PLM samples), and with no graminaceous stomata observed, we conservatively avoid assigning these phytolith groups to any specific plants.

Elongate dendritic phytoliths are formed in Poaceae (Parry and Smithson, 1966; Strömberg, 2003), Cyperaceae, Arecaceae, and Marantaceae (ICPN 2.0, 2019). Strömberg (2003) also reports them from several dicots. We observed a substantial amount of these silica bodies in all our samples, along with many transitional forms to elongate entire, elongate sinuate, and elongate dentate types, revealing no significant correlation or difference in these subtypes.

Tracheary annulate/helical phytoliths (**Figure 4R**) are another relatively common group of phytoliths found in nearly equal amounts in fresh, dry, herbarized palm leaves, and manuscript samples. In *Corypha* samples, these occurred more frequently. These phytoliths are reported from a wide range of plants, including Arecaceae (confirmed by our study), Poaceae (Strömberg, 2003), gymnosperms (Wallis, 2003; Piperno, 2006), and conifers (e.g., Klein and Geis, 1978).

### 4.3 Non-Arecaceae phytolith in the unprocessed palm-leaf material

About ca 5% of TPS in the fresh, dry and herbarized palm-leaf material of *Borassus* and *Corypha* belong to non-Arecaceae phytoliths (**Figures 5**, **7**–**9**; **Table 2**). Despite it is usually agreed that in the environmental reconstructions based e.g., on soils and sediments, the role of long-distance wind component of phytoliths is small, as phytolith material is usually not dispersed over the regional distance scale by wind in any considerable amount (Åkesson et al., 2021), we cannot completely deny a role of random dust contamination (See Latorre et al., 2012). As small phytoliths are often found in dust, and dust is in most cases unavoidable in nature, some ‘foreign’ phytoliths should be expected in all types of the studied material regardless of their origin. However, in our samples their amounts demonstrated a fluctuation at around 4% for unprocessed palm leaf samples of the both studied species, never exceeding 4.8% (in fresh leaf material of *Corypha*). This suggests that random phytolith contamination for palm material is relatively stable and small. Obviously, samples taken from herbaria and other long-term stored collections, especially the old samples taken from the objects kept in the collection since 1950 and earlier, tend to bear more mineral dust containing the non-Arecaceae (and non-Coriphoideae) phytoliths. Some unmeasurable contamination can be also inserted by the regular handling of the collections. Still, these contaminants do not seem to play any important role in the TPS, as it follows from our diagrams (**Figures 5**, **7**–**9** with the non-diagnostic phytoliths profiled separately regardless of their origin). Furthermore, ordination did not reveal any differences between the higher and lower contaminated samples of the unprocessed leaf material of the both species; their statistical errors stay within 1SD, which is well comparable with other samples. That allows to conclude that the random environmental contamination of the palm-leaf material cannot compromise the overall results and their interpretation of the phytolith analysis to any statistically significant extend.

### 4.4 Geographical differences in the phytolith assemblages described from PLMs

A considerable number of phytoliths of cf. *Azadirachta indica* (5%; **Figures 5**, **7**) in CM-11 cannot be an artifact or misidentification, given their distinct morphology. Neem phytoliths have a roundish form with a more flattened and granulate surface (65% of all analyzed silica bodies of *Azadirachta indica* in this study; **Figure 4J**). Neem grows in the low-lying northern and eastern parts of Java and in the eastern isles, including Bali and Lombok (Saxena et al., 1993). In Bali, Hinduism, some similar to India, is practiced (Picard and Ramstedt, 2004), suggesting similar rituals and worship. Alternatively, neem phytoliths on the surface of Balinese manuscript CM-11 could result from later conservation treatments (e.g., cleaning with neem leaves or fumigation with burned leave material).

The use of rice was identified in *Borassus* PLMs (BM-1, BM-2, BM-3, BM-11, BM-13, BM-18), originating from Tamil Nadu, India, and BM-18 from Bali. In *Corypha* manuscripts, rice use was evident in CM-1 (Sri Lanka), CM-8 (Kerala, India), CM-13, and CM-18 (Tamil Nadu, India). For all these sites, literature mentions rice used for seasoning, brushing, and polishing manuscripts. Other Poaceae might also have been utilized in different regions. Use of *Dendrocalamus* sp. as firewood was possible during the production process of BM-18 (Bali, Indonesia) and BM-20, BM-22 (Tamil Nadu, India). Leaves of woody bamboos were likely used for cleaning Singhalese CM-1, explaining its distinct position in ordination graphs (**Figures 10F**, **11**). Analysis of more Sri Lankan material was limited due to sample size (CM-1 and CM-12). More material is required for better interpretation.

*Zizania* sp. was identified in three PLMs from Burma/Myanmar (BM-17, CM-9, and CM-10), though statistical analysis did not form a separate group, likely due to the small sample size and limited differentiation from other manuscripts. This indicates a unique aspect of Burmese PLM production compared to neighboring regions.

We were unable to distinguish PLM material from Kerala (India) using current phytolith analysis, as PLMs from Kerala (CM-2, CM-7) grouped with other Indian samples, and CM-8 ordinated close to CM-13 (Tamil Nadu) due to similar rice phytolith percentages. Literature suggests most plants used in Kerala produce few or no phytoliths (Piperno, 2006; Alahakoon, 2012; Nishanthi and Wijayasundara, 2022), including *Carica papaya*, *Capsicum* sp., *Illicium verum*, *Nicotiana* sp., and *Cinnamomum* sp (Padmakumar and Sreekumar, 2003; Sah, 2006; Perumal, 2013). Identification of *Vitex* sp. based on phytolith analysis was not feasible, and DNA analysis is recommended for accurate identification of these plants. In summary, PCA analysis well reflect the geographical origins of the samples based on associated plants used during manuscript creation rather than the palm species. Geographic origin is a more significant indicator of phytolith diversity than taxonomic classification of the writing support material. The correlation of Arecaceae phytoliths with most *Corypha* samples is an artifact resulting from the widespread presence of palm spheroids in all samples.

### 4.5 Problems of phytolith analysis from PLMs and challenges in its interpretation

As demonstrated in our study on the PLM material of *Borassus flabellifer* and *Corypha umbraculifera*, there is a clear, statistically significant, and stable difference between the phytolith assemblages from the manuscripts and other studied material. This was proven both qualitatively and quantitatively by comparing freshly collected, dried, and herbarized material of the same species. The investigated phytolith assemblages appear more dependent on the set and amounts of plants used in the PLM production rather than on the palm species used as manuscript writing supports. This difference includes geographical variability, though more research is needed in this area.

Palaeoecological studies of PLMs offer a new perspective for gaining knowledge on their production and handling history. This approach combines methods of applied humanities and natural sciences to reconstruct the creation recipes of written artifacts and possibly reveal their geographical origins. Even without a manuscript’s colophon, its text, script, and language can suggest probable geographical origins. Reconstruction practices based on plant proxies, such as phytoliths, charcoal particles, and potentially ancient plant DNA, can answer material codicology questions and expand application of traditional plant proxy-based palaeoecological methods. This research proposes using these methods in studying the material history and provenance of manuscripts. However, the future application of this methodology presents several problems and limitations.

#### 4.5.1 Multiplicity and redundancy

Phytoliths are produced in different plant parts, resulting in variations in shapes and morphotypes (Rapp and Mulholland, 1992; Ball et al., 1993). This phenomenon, termed multiplicity (Rovner, 1971), complicates identifying source plants. Similar morphotypes may be produced by different taxa, not necessarily closely related, leading to redundancy (Rovner, 1971). For example, spheroid echinate morphotypes can be found in palms (Morcote-Ríos et al., 2016; Witteveen et al., 2022; this study), pineapple (Ferreira and de Araujo, 2010; Corrales-Ureña et al., 2018), plants of e.g., Zingiberaceae, Anacardiaceae, Orchidaceae families (Kealhofer and Piperno, 1998; Benvenuto et al., 2015). In addition, phytolith types like blocky, elongate entire, elongate sinulate, tracheary have low taxonomic value (ICPN 2.0, 2019). In order to avoid overinterpretation, a conservative approach with good reference collections and regional studies on phytolith morphology is recommended.

#### 4.5.2 Low or no phytolith production in some plants

Some plants important in PLM production, such as those from Fabaceae, Malvaceae, and Piperaceae, produce few phytoliths, while others, such as those from Apiaceae and Rutaceae, produce non-diagnostic phytoliths only (Piperno, 2006). Plants like *Carica papaya* and *Capsicum* sp. do not produce phytoliths (Piperno, 2006). Lemon grass (*Cymbopogon* sp.) often reported to be used for seasoning, oiling, conservation, cleaning and increasing attractiveness of the manuscripts (e.g., Sah, 2002; Sahoo and Mohanty, 2007; Alahakoon, 2012) can only be identified in a complex with other Poaceae. For the same reason, the possibility to clearly identify castor beans (*Ricinus communis*; Sah, 2002), cinnamon (*Cinnamonum zeylanicumi*; Sah, 2006), black thorn apple (*Datura stramonium*; Sah, 2002; Perumal, 2013; Wilson and Rice, 2019; Sharma et al., 2021), *Faba* sp./*Vicea faba* (Bisoi, 1995; Sah, 2002; Meher, 2009), indigo leaves and bark (*Indigofera tinctorial*; Sah, 2002; Meher, 2009; Sharma et al., 2021) purely on the basis of phytolith analysis is doubtful.

#### 4.5.3 Depositional histories

As it was discussed by Vrydaghs et al. (2016), in applied soil and archaeological phytolith studies, phytoliths not only come from different taxa, but they can be sourced from the different depositional histories (Shillito, 2011). This aspect of the phytoliths taphonomy is also relevant for the studies of PLMs: we may well expect material sedimented on the surface of the PLMs at different times and during the various steps of the preparation process. The accumulation layers can be extremely thin, discontinuous, interrupted, mixed up or even partly removed, e.g., because of cleaning or just because of the active handling.

#### 4.5.4 Contamination

Random contamination resulting from handling manuscripts, including food stains, finger oils, various ashes, paints, soils, sediments, blood, pollen, spores, bacteria, and other agents, can affect phytolith assemblages on PLM surfaces. However, modern inorganic dust contamination does not significantly influence the results, as we demonstrated in this study.

#### 4.5.5 Ethical considerations

PLMs are unique and valuable parts of the global cultural heritage. Invasive sampling can be harmful to manuscripts if applied carelessly, and may conflict with conservation and preservation efforts. Developing minimally invasive manuscript material sampling methods is crucial. While the application of phytolith analysis in PLM studies is promising, careful consideration of these challenges and constraints is necessary to ensure accurate and ethical research outcomes.

## 5 Summary and concluding remarks

The creation of palm-leaf manuscripts (PLMs) involves the use of various plants, and both these plants and practices can be region-specific. Knowing this from the literature, we applied phytolith analysis to fresh, dry, and herbarized palm leaf material of two palm species, *Borassus flabellifer* L. and *Corypha umbraculifera* L., commonly used for PLM production in S and SE Asia. Microsamples of 50 PLMs originating from the Indian states of Kerala (4 samples) and Tamil Nadu (36 samples), from Sri Lanka (2 samples), Burma/Myanmar (3 samples), and Indonesia (Lambok and Bali islands, 5 samples) were analyzed. This research revealed that variability in phytolith assemblages did not depend on the palm species used as manuscript support. Geographic origin is a more significant indicator of phytolith diversity than taxonomic classification of the writing support material. Natural contamination of the research material was consistent across all analyzed samples, fluctuating around 4% and never exceeding 5%, which does not notably influence phytolith analysis results. Phytolith assemblages from PLMs highly vary; this difference is potentially useful in future studies of PLM production processes. This approach can help identify plants involved in PLM production across different historical periods and geographical regions. It offers a perspective to reconstruct ancient, poorly described and undescribed PLM creation recipes, contributing to understanding local cultural practices and plant use customs over time and space. This methodology may also retrieve the geographical origin of palm-leaf written artifacts with unclear or unknown provenance, possibly aiding in resolving issues of looted artifacts and distinguishing fake artifacts. These interpretations, however, should be approached with caution due to the contradictory, scarce, and sometimes unreliable information on PLM production practices. More studies are needed on phytoliths from local and regional plants in S and SE Asia and on PLM production practices within the region. Understanding the historical trends of palm-leaf material trading is crucial, as leaves may have been prepared in one place and exported to another. Efforts should focus on revealing possible constraints and limitations of the proposed approach.

## Nomenclature

The phytolith morphology and terminology is based on the International Code for Phytolith Nomenclature (ICPN; Neumann et al., 2019). Plant taxonomy is followed the Angiosperm Phylogeny Group (APG 2016).

## Data Availability

The datasets analyzed for this study can be found in the Zenodo repository: https://zenodo.org/uploads/12773459.

## Supplementary material

Supplementary Material S1 Samples of fresh and dry leaves of *Borassus flabellifer* and *Corypha umbraculifera* collected for phytolith studies in 2022-2024.

Supplementary Material S2 Herbarium samples of *Borassus flabellifer* and *Corypha umbraculifera* collected for phytolith studies in 2022-2024.

Supplementary Material S3 Complete list of all phytolith types and inorganic crystals registered in the palm leave material of *Borassus flabellifer* and *Corypha umbraculifera* with indication of their presence in the fresh, dry, herbarized and manuscript samples of two palm species.

Supplementary Material S4 Complete phytolith diagram for palm leave material of *Borassus flabellifer.*

Supplementary Material S5 Complete phytolith diagram for palm leave material of *Corypha umbraculifera.*

Supplementary Material S6 PCA scores for ordination of the palm leave material of *Borassus flabellifer.*

Supplementary Material S7 PCA scores for ordination of the palm leave material of *Corypha umbraculifera.*

Supplementary Material S8 PCA scores for ordination of the palm-leave manuscript material of *Borassus flabellifer* and *Corypha umbraculifera.*
