# Supplementary material for: Phytolith assemblages from palm leaves and palm-leaf manuscripts: what is the difference and what it could mean?
Source: Front Plant Sci. 2025 Jan 14;15:1482790. doi: 10.3389/fpls.2024.1482790 (PMC11772424; doi:10.3389/fpls.2024.1482790)
Supplement: Supplementary Material S1 — Samples of fresh and dry leaves of Borassus flabellifer and Corypha umbraculifera collected for phytolith studies in 2022-2024. [file Table1.docx]

Table S1. Samples of fresh and dry leaves of *Borassus flabellifer* and *Corypha umbraculifera* collected for phytolith studies in 2022-2024. TN stays for Tamil Nadu; KRL stays for Kerala state of India.

| *Borassus flabellifer* | | | | | | | |
| --- | --- | --- | --- | --- | --- | --- | --- |
| Sample number | Sample code | Collection site description | Latitude, N | Longitude, E | Elevation, m asl. | Collecting date |  |
| BF-1/BD-1 | TN_BO_002 | Villiyanallur, Tamil Nadu (mature leaf) | 11°31.349' | 79°43.169' | 8 | 02/05/2024 |  |
| BF-2/BD-2 | TN_BO_005 | Thirukadaiyur, Tamil Nadu (mature leaf) | 11°4'46.5492'' | 79°48'10.3716'' | 7.5 | 02/05/2024 |  |
| BF-3/BD-3 | TN_BO_007 | Keezhaiyur, South Vanjur, Nagor Road, Karaikal (young leaf) | 10°50'29.0616'' | 79°49'55.02'' | 4 | 03/05/2024 |  |
| BF-4/BD-4 | TN_BO_007 | Keezhaiyur, South Vanjur, Nagor Road, Karaikal (mature leaf) | 10°50'29.0616'' | 79°49'55.02'' | 4 | 03/05/2024 |  |
| BF-5/BD-5 | TN_BO_010 | Light House Road Kodiakkadu, Kodiyakari, Tamil Nadu | 10°17'05.3" | 79°49'34.8" | 6 | 04/05/2024 |  |
| BF-6/BD-6 | TN_BO_011 | Kodiakkadu, Tamil Nadu (young leaf) | 10°17'50.1" | 79°49'19.3" | 6 | 04/05/2024 |  |
| BF-7/BD-7 | TN_BO_011 | Kodiakkadu, Tamil Nadu (mature leaf) | 10°17'50.1" | 79°49'19.3" | 6 | 04/05/2024 |  |
| BF-8/BD-8 | TN_BO_012 | Salt Pan, Kodiyakarai, Tamil Nadu (mature leaf) | 10°21'17.2" | 79°50'26.5" | 5 | 04/05/2024 |  |
| BF-9/BD-9 | TN_BO_015 | Anniyur, Tamil Nadu (young leaf) | 10°57'59.0" | 79°34'02.6" | 75 | 05/05/2024 |  |
| BF-10/BD-10 | TN_BO_015 | Anniyur, Tamil Nadu (mature leaf) | 10°57'59.0" | 79°34'02.6" | 75 | 05/05/2024 |  |
| BF-11/BD-11 | TN_BO_016 | Papireddipatti, Tamil Nadu, Kumbakonam-Tanjavur Road (young leaf) | 10°55'59.2" | 79°17'00.9" | 41 | 05/05/2024 |  |
| BF-12/BD-12 | TN_BO_016 | Papireddipatti, Tamil Nadu, Kumbakonam-Tanjavur Road (mature leaf) | 10°55'59.2" | 79°17'00.9" | 410 | 05/05/2024 |  |
| BF-13/BD-13 | TN_BO_017 | Pasupattikoil, Tamil Nadu (young leaf) | 10°52'20.0 | 79°10'25.9" | 39 | 05/05/2024 |  |
| BF-14/BD-14 | TN_BO_017 | Pasupattikoil, Tamil Nadu (mature leaf) | 10°52'20.0 | 79°10'25.9" | 39 | 05/05/2024 |  |
| BF-15/BD-15 | TN_BO_018 | Vayaloor, Narasanayagipuram, Tamil Nadu (young leaf) | 10°50'53.3" | 79°09'15.6" | 325 | 05/05/2024 |  |
| BF-16/BD-16 | TN_BO_018 | Vayaloor, Narasanayagipuram, Tamil Nadu (mature leaf) | 10°50'53.3" | 79°09'15.6" | 325 | 05/05/2024 |  |
| BF-17/BD-17 | TN_BO_019 | Vallam Pudursethi, Tamil Nadu  (young leaf) | 10°43'38.5" | 79°02'59.0" | 75 | 05/05/2024 |  |
| BF-18/BD-18 | TN_BO_019 | Vallam Pudursethi, Tamil Nadu (mature leaf) | 10°43'38.5" | 79°02'59.0" | 75 | 05/05/2024 |  |
| BF-19/BD-19 | TN_BO_AP1 | Arulmigy, Tamil Nadu (mature leaf) | 12°39'22.84" | 79°57'22.66" | 213 | 26/06/2024 |  |
| BF-20/BD-20 | TN_BO_AP2 | Arulmigy, Tamil Nadu (mature leaf) | 12°39'22.72" | 79°57'22.79" | 213 | 26/06/2024 |  |
| BF-21/BD-21 | TN_BO_AP3 | Arulmigy, Tamil Nadu (mature leaf) | 12°39'22.77" | 79°57'22.48" | 213 | 26/06/2024 |  |
| BF-22/BD-22 | TN_BO_AP4 | Arulmigy, Tamil Nadu (mature leaf) | 12°39'22.56" | 79°57'22.74" | 213 | 26/06/2024 |  |
| BF-23/BD-23 | TN_BO_AP5 | Arulmigy, Tamil Nadu (mature leaf) | 12°39'22.63" | 79°57'22.51" | 211 | 26/06/2024 |  |
| BF-24/BD-24 | TN_BO_AP6 | Arulmigy, Tamil Nadu (mature leaf) | 12°39'22.54" | 79°57'22.62" | 211 | 26/06/2024 |  |
| BF-25/BD-25 | TN_BO_AP7 | Arulmigy, Tamil Nadu (mature leaf) | 12°39'22.53" | 79°57'22.52" | 211 | 26/06/2024 |  |
| *Corypha umbraculifera* | | | | | | | |
| CF-1/CD-1 | TN_CO_006a | Polagam, Kaliamman Kovil Theru, Karaikal, Puducherry | 10°51'42.8" | 79°49'17.8" | 6 | 03/05/2024 |  |
| CF-2/CD-2 | TN_CO_009 | Nallichery, Tamil Nadu | 10°57'56.6" | 79°34'04.8" | 20 | 05/05/2024 |  |
| CF-3/CD-3 | TN_CO_010 | Kudavasal, Tamil Nadu | 10°56'34.0" | 79°34'37.9" | 45 | 05/05/2024 |  |
| CF-4/CD-4 | TN_CO_010 | Kudavasal, Tamil Nadu | 10°56'34.0" | 79°34'37.9" | 45 | 05/05/2024 |  |
| CF-5/CD-5 | TN_CO_011 | Vayaloor, Narasanayagipuram, Tamil Nadu | 10°50'53.3" | 79°09'15.6" | 60 | 05/05/2024 |  |
| CF-6/CD-6 | TN_CO_012 | Vallam Pudursethi, Tamil Nadu | 10°43'38.5" | 79°02'59.0" | 75 | 05/05/2024 |  |
| CF-7/CD-7 | KRL_CO_AP1 | Santo Nagar – field side, Kerala | 10°13'4.17" | 76°22'53.64" | 600 | 05/07/2024 |  |
| CF-8/CD-8 | KRL_CO_AP2 | Santo Nagar – field side, Kerala | 10°13'3.91" | 76°22'54.49" | 600 | 05/07/2024 |  |
| CF-9/CD-9 | KRL_CO_AP3 | Santo Nagar – field side, Kerala | 10°13'3.65" | 76°22'53.99" | 600 | 05/07/2024 |  |
| CF-10/CD-10 | KRL_CO_AP4 | Santo Nagar – field side, Kerala | 10°13'3.63" | 76°22'55.48" | 600 | 05/07/2024 |  |
| CF-11/CD-11 | KRL_CO_AP5 | Santo Nagar – field side, Kerala | 10°13'4.70" | 76°22'56.44" | 600 | 05/07/2024 |  |
| CF-12/CD-12 | KRL_CO_AP6 | Santo Nagar – field side, Kerala | 10°13'4.45" | 76°22'56.78" | 600 | 05/07/2024 |  |
| CF-13/CD-13 | KRL_CO_AP7 | Santo Nagar – road side, Kerala | 10°13'6.17" | 76°22'41.27" | 400 | 05/07/2024 |  |
| CF-14/CD-14 | KRL_CO_AP8 | Santo Nagar – road side, Kerala | 10°13'5.85" | 76°22'41.73" | 400 | 05/07/2024 |  |
| CF-15/CD-15 | KRL_CO_AP9 | Kuluthu Kavu, Kerala | 10°22'57.37" | 76°17'43.64" | 64 | 05/07/2024 |  |
| CF-16/CD-16 | KRL_CO_AP10 | Kuluthu Kavu, Kerala | 10°22'56.89" | 76°17'44.32" | 64 | 05/07/2024 |  |
| CF-17/CD-17 | KRL_CO_AP11 | Kuluthu Kavu, Kerala | 10°22'56.74" | 76°17'43.76" | 64 | 05/07/2024 |  |
| CF-18/CD-18 | KRL_CO_AP12 | Kuluthu Kavu, Kerala | 10°22'56.50" | 76°17'44.34" | 64 | 05/07/2024 |  |
| CF-19/CD-19 | KRL_CO_AP13 | Pudukad Grama Panchayhath, Kerala | 10°25'32.01" | 76°16'8.42" | 20 | 05/07/2024 |  |
| CF-20/CD-20 | KRL_CO_AP14 | Pudukad Grama Panchayhath, Kerala | 10°25'32.19" | 76°16'8.18" | 20 | 05/07/2024 |  |
| CF-21/CD-21 | KRL_CO_AP15 | Pudukad Grama Panchayhath, Kerala | 10°25'31.95" | 76°16'8.92" | 20 | 05/07/2024 |  |
| CF-22/CD-22 | KRL_CO_AP16 | Pudukad Grama Panchayhath, Kerala | 10°25'32.17" | 76°16'8.70" | 20 | 05/07/2024 |  |
| CF-22/CD-23 | KRL_CO_AP17 | Pudukad Grama Panchayhath, Kerala | 10°25'31.97" | 76°16'9.18" | 20 | 05/07/2024 |  |
| CF-24/CD-24 | KRL_CO_AP18 | Pudukad Grama Panchayhath, Kerala | 10°25'31.87" | 76°16'9.13" | 20 | 05/07/2024 |  |
| CF-25/CD-25 | KRL_CO_AP19 | Pudukad Grama Panchayhath, Kerala | 10°25'32.04" | 76°16'9.46" | 20 | 05/07/2024 |  |
