# Supplementary material for: Phytolith assemblages from palm leaves and palm-leaf manuscripts: what is the difference and what it could mean?
Source: Front Plant Sci. 2025 Jan 14;15:1482790. doi: 10.3389/fpls.2024.1482790 (PMC11772424; doi:10.3389/fpls.2024.1482790)
Supplement: Supplementary Material S2 — Herbarium samples of Borassus flabellifer and Corypha umbraculifera collected for phytolith studies in 2022-2024. [file Table2.docx]

Table S2. Herbarium samples of *Borassus flabellifer* and *Corypha umbraculifera* collected for phytolith studies in 2022-2024. “?” stays when the year is uncertain (unclearly written on the herbarium label).

| *Borassus flabellifer* | | | | |
| --- | --- | --- | --- | --- |
| Sample number | Sample code and original code of herbarium specimens if known | Name of herbarium collection / Plant material origin | Age of specimens, yrs | Sampling date |
| BH-1 | GOETO_025089 (leaf 1) | GOET / Botanic Garden Malabar, India | 1879 | 20.01.2022 |
| BH-2 | GOETO_025089 (leaf 2) | GOET / Botanic Garden Malabar, India | 1879 | 20.01.2022 |
| BH-3 | Kew-084-2023 (unattached fragment) | Kew Gardens / India, Tamil Nadu | 1981 | 02.06.2023 |
| BH-4 | Kew-084-2023 (leaf 1) | Kew Gardens / India, Tamil Nadu | 1981 | 02.06.2023 |
| BH-5 | Kew-085-2023 (unattached fragment 1) | Kew Gardens / India | 1922 ? | 02.06.2023 |
| BH-6 | Kew-085-2023 (unattached fragment 2) | Kew Gardens / India | 1922 ? | 02.06.2023 |
| BH-7 | Kew-086-2023 (leaf 1) | Kew Gardens / India, Calcutta | 1901 | 02.06.2023 |
| BH-8 | Kew-086-2023 (leaf 2) | Kew Gardens / India, Calcutta | 1901 | 02.06.2023 |
| BH-9 | Kew-087-2023 (unattached fragment) | Kew Gardens / India (now Bangladesh), Chittagong | 1867 | 02.06.2023 |
| BH-10 | Kew-087-2023 (leaf 1) | Kew Gardens / India (now Bangladesh), Chittagong | 1867 | 02.06.2023 |
| BH-11 | Kew-088-2023 (unattached fragment) | Kew Gardens / Sri Lanka (Ceylon) | 1993 | 02.06.2023 |
| BH-12 | Kew-088-2023 (leaf 1) | Kew Gardens / Sri Lanka (Ceylon) | 1993 | 02.06.2023 |
| BH-13 | Kew-089-2023 K001098622 (leaf 1) | Kew Gardens / Indo-China (Siam, now Thailand) | 1859 | 02.06.2023 |
| BH-14 | Kew-089-2023 K001098622 (unattached fragment 1) | Kew Gardens / Indo-China (Siam, now Thailand) | 1859 | 02.06.2023 |
| BH-15 | Kew-089-2023 K001098622 (unattached fragment 2) | Kew Gardens / Indo-China (Siam, now Thailand) | 1859 | 02.06.2023 |
| BH-16 | Kew-090-2023 K001098623 (leaf 1) | Kew Gardens / Thailand, Bangkok | 1926 | 02.06.2023 |
| BH-17 | Kew-090-2023 K001098623 (unattached fragment 1) | Kew Gardens / Thailand, Bangkok | 1926 | 02.06.2023 |
| BH-18 | Kew-090-2023 K001098623 (unattached fragment 2) | Kew Gardens / Thailand, Bangkok | 1926 | 02.06.2023 |
| BH-19 | Kew-091-2023 K001098616 (leaf 1) | Kew Gardens / Thailand | 2002 | 05.06.2023 |
| BH-20 | Kew-091-2023 K001098616 (leaf 2) | Kew Gardens / Thailand | 2002 | 05.06.2023 |
| BH-21 | Kew-091-2023 K001098616 (leaf 3) | Kew Gardens / Thailand | 2002 | 05.06.2023 |
| BH-22 | Kew-092-2023 K001098617 (leaf 1) | Kew Gardens / Malaysia, Kelantan | 1937 | 05.06.2023 |
| BH-23 | Kew-092-2023 K001098617 (leaf 2) | Kew Gardens / Malaysia, Kelantan | 1937 | 05.06.2023 |
| BH-24 | Kew-141-2023 K001098620 (leaf 1) | Kew Gardens / Botanic Garden, Peradeniya, Sri Lanka | 1986 | 06.06.2023 |
| BH-25 | Kew-141-2023 K001098620 (leaf 2) | Kew Gardens / Botanic Garden, Peradeniya, Sri Lanka | 1986 | 06.06.2023 |
| *Corypha umbraculifera* | | | | |
| CH-1 | GOETO_025482 (leaf 1) | GOET / Botanic Garden Calcutta, India | 1911 | 20.01.2022 |
| CH-2 | GOETO_025482 (leaf 2) | GOET / Botanic Garden Calcutta, India | 1911 | 20.01.2022 |
| CH-3 | Kew-001-2023 (unattached fragment) | Kew Gardens / Sri Lanka (Ceylon) | 1867 | 30.05.2023 |
| CH-4 | Kew-001-2023 (leaf 1) | Kew Gardens / Sri Lanka (Ceylon) | 1867 | 30.05.2023 |
| CH-5 | Kew-001-2023 (leaf 2) | Kew Gardens / Sri Lanka (Ceylon) | 1867 | 30.05.2023 |
| CH-6 | Kew-021-2023 / K000521704 (leaf 1) | Kew Gardens / Kew Gardens Tropical greenhouse  (originally from India) | 1998 | 31.05.2023 |
| CH-7 | Kew-021-2023 / K000521704 (leaf 2) | Kew Gardens / Kew Gardens Tropical greenhouse  (originally from India) | 1998 | 31.05.2023 |
| CH-8 | Kew-021-2023 / K000521704 (unattached fragment) | Kew Gardens / Kew Gardens Tropical greenhouse  (originally from India) | 1998 | 31.05.2023 |
| CH-9 | Kew-023-2023 / K001083525 (unattached fragment 1) | Kew Gardens / Sri Lanka (Ceylon) | 1986 | 31.05.2023 |
| CH-10 | Kew-023-2023 / K001083525 (unattached fragment 2) | Kew Gardens / Sri Lanka (Ceylon) | 1986 | 31.05.2023 |
| CH-11 | Kew-023-2023 / K001083525 (unattached fragment 3) | Kew Gardens / Sri Lanka (Ceylon) | 1986 | 31.05.2023 |
| CH-12 | Kew-023-2023 / K001083525 (leaf 1) | Kew Gardens / Sri Lanka (Ceylon) | 1986 | 31.05.2023 |
| CH-13 | Kew-024-2023 (leaf 1) | Kew Gardens / Yunnan Institute of tropical botanical culture, Botanic Garden, China | 1978 | 31.05.2023 |
| CH-14 | Kew-024-2023 (leaf 2) | Kew Gardens / Yunnan Institute of tropical botanical culture, Botanic Garden, China | 1978 | 31.05.2023 |
| CH-15 | Kew-024-2023 (unattached fragment) | Kew Gardens / Yunnan Institute of tropical botanical culture, Botanic Garden, China | 1978 | 31.05.2023 |
| CH-16 | Kew-025-2023 / K001083524 (unattached fragment 1) | Kew Gardens / Botanic Garden of Singapore | 1936 | 31.05.2023 |
| CH-17 | Kew-025-2023 / K001083524 (unattached fragment 2) | Kew Gardens / Botanic Garden of Singapore | 1936 | 31.05.2023 |
| CH-18 | Kew-025-2023 / K001083524 (leaf 1) | Kew Gardens / Botanic Garden of Singapore | 1936 | 31.05.2023 |
| CH-19 | Kew-026-2023 (unattached fragment 1) | Kew Gardens / Botanic Garden Miami, Florida, USA (originally from India) | 1984 | 31.05.2023 |
| CH-20 | Kew-026-2023 (unattached fragment 2) | Kew Gardens / Botanic Garden Miami, Florida, USA  (originally from India) | 1984 | 31.05.2023 |
| CH-21 | Kew-026-2023 (unattached fragment 3) | Kew Gardens / Botanic Garden Miami, Florida, USA  (originally from India) | 1984 | 31.05.2023 |
| CH-22 | Kew-030-2023 / K001098285 (leaf 1) | Kew Gardens / Sri Lanka (Ceylon) | Mid. XX cent. | 31.05.2023 |
| CH-23 | Kew-030-2023 / K001098285 (leaf 2) | Kew Gardens / Sri Lanka (Ceylon) | Mid. XX cent. | 31.05.2023 |
| CH-24 | Kew-030-2023 / K001098285 (unattached fragment 1) | Kew Gardens / Sri Lanka (Ceylon) | Mid. XX cent. | 31.05.2023 |
| CH-25 | Kew-030-2023 / K001098285  (unattached fragment 2) | Kew Gardens / Sri Lanka (Ceylon) | Mid. XX cent. | 31.05.2023 |
