# Supplementary figures and images for: Phytolith assemblages from palm leaves and palm-leaf manuscripts: what is the difference and what it could mean?
Source: Front Plant Sci. 2025 Jan 14;15:1482790. doi: 10.3389/fpls.2024.1482790 (PMC11772424; doi:10.3389/fpls.2024.1482790)

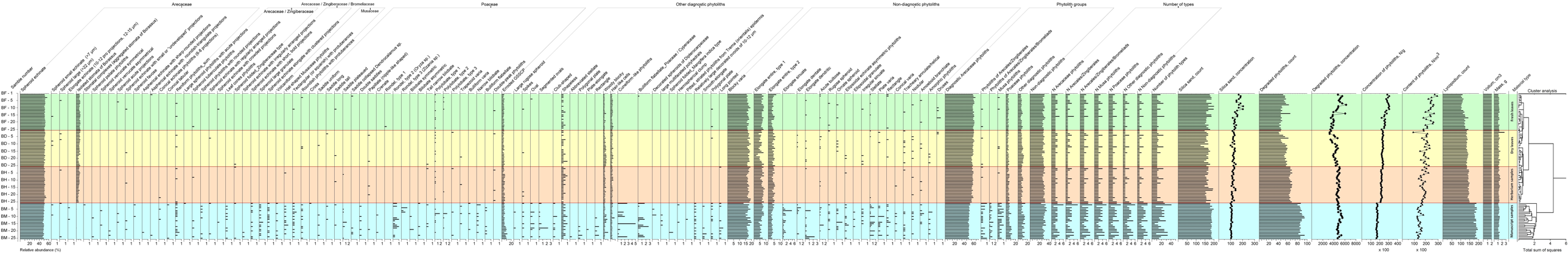

Supplement: Supplementary Material S8 — PCA scores for ordination of the palm-leave manuscript material of Borassus flabellifer and Corypha umbraculifera. [file Image2.jpeg]
